# From amines to (form)amides: a simple and successful mechanochemical approach

**DOI:** 10.3762/bjoc.18.126

**Published:** 2022-09-12

**Authors:** Federico Casti, Rita Mocci, Andrea Porcheddu

**Affiliations:** 1 Dipartimento di Scienze Chimiche e Geologiche, Università degli Studi di Cagliari, Cittadella Universitaria, 09042 Cagliari, Italy

**Keywords:** acetamides, formamides, mechanochemistry, *N*-formylation, *p*-tosylimidazole

## Abstract

Two easily accessible routes for preparing an array of formylated and acetylated amines under mechanochemical conditions are presented. The two methodologies exhibit complementary features as they enable the derivatization of aliphatic and aromatic amines.

## Introduction

The preparation of *N*-formylated and *N*-acetylated amines plays a crucial role in organic synthesis [1–6]. In one respect, it is relevant to protect the amine group straightforwardly and under mild conditions [7–8]. On the other hand, the formamide and acetamide moieties are found in many active pharmaceutical ingredients (APIs) and natural products [1,9]. *N*-Formyl derivatives were used as building blocks in Vilsmeier–Haack reactions [10–11] and for preparing molecule drug substances, various heterocycles, formamidines, isocyanates, and isocyanides [12–17]. The large number of procedures reported in the literature witnesses the relevance of this class of compounds [9,18–26]. However, despite the remarkable advancement in the field, many conventional methodologies proceed at high temperatures and require an expensive catalyst and toxic reagents. In addition, these procedures only work in substantial excess of formyl or acyl sources, often used as a solvent.

Mechanochemistry has been established as a powerful tool for the rapid, clean, and environmentally friendly synthesis of organic compounds, avoiding bulk solvent and restrictions of solvent-based chemistry [27–34]. In general, mechanochemistry refers to studying solid-state chemical changes promoted by external mechanical energy, such as grinding or milling. A deeper understanding of its mechanistic aspects laid the basis for further growth in this topic, opening new routes to more efficient mechanochemical reactions [35–42].

In our effort to develop green and sustainable methodologies using mechanochemistry [43–46], we recently studied a protocol for synthesizing isocyanides using *p*-tosyl chloride (Ts-Cl) in basic conditions, starting from the corresponding formamides [47]. In this work, we aimed to set compatible conditions to access formamides, envisioning the possibility of generating the isocyanide in a one-pot, two-step reaction. However, to the best of our knowledge, despite the notable improvement in the mechanochemical synthesis of the amide moiety [44,48–54], no systematic report of *N*-formylation and *N*-acetylation by ball milling has been reported yet. Here, we describe two complementary procedures to prepare formamides and acetamides, applied to primary and secondary aromatic and aliphatic amines. The methodologies directly involve HCO_2_H derivatives and CH_3_CO_2_H and two activating agents for promoting amide coupling.

## Results and Discussion

We started our investigation by reacting *p*-methoxyaniline (1.0 mmol) with ammonium formate (3.0 mmol) (Table 1, entry 1) in a zirconia jar in the presence of one milling ball of the same material (Ø = 8 mm, *m* = 3.2 g) [22,55] in a horizontal vibratory mill at 30 Hz. Under these conditions, we did not detect the formation of the formamide moiety. At the same time, the desired product **2** was obtained in 16% NMR yield when formic acid was added to the reaction mixture (Table 1, entry 2). Switching to sodium sulfate as a grinding additive significantly enhanced the reaction performance (Table 1, entries 3 and 4). At this stage, we wondered, if using a more efficient dehydrating agent would be mandatory for the reaction to occur. Therefore, we turned our attention toward *p*-tosylimidazole *(p*-Ts-Im), a cheap and commercially available reagent directly prepared from *p*-toluensulfonic acid by reaction with 1,1′-carbonyldiimidazole (CDI). The compound proved very effective for dehydrating oximes under mechanochemical acidic Beckmann conditions [44]. Moreover, it represents a suitable and compatible means in the view of a one-pot methodology for preparing isocyanides directly from amines [56].

**Table 1:** Optimization of reaction conditions for **2**.^a,b^.

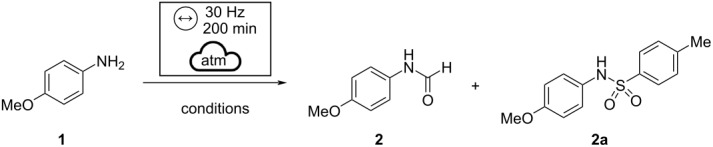				

Entry	Formic acid (equiv)	Additives (equiv)	Yield of **2** (%)^c^	Ratio **2**:**2a**

1	–	HCOONH_4_ (3.0)	–	–
2	1.5	HCOONH_4_ (3.0)	16	–
3	1.5	Na_2_SO_4_ (2.8)	40	–
4	1.5	Na_2_SO_4_ (2.8)/ MeOH (LAG, η = 0.2)	45	–
5	1.5	*p*-Ts-Im/Et_3_N 1:1	56	70:30
6	2	*p*-Ts-Im (1.0)	75	90:10
7^d^	2	*p*-Ts-Im (1.0)	85	88:12
8^d^	2	*p*-Ts-Im (0.1)	85	95:5
**9** ^d^	**2**	**imidazole (1.0)**	**94**	–

^a^The reaction scheme was depicted using the symbolism proposed in [57]. ^b^Conditions: compound **1** (1.0 mmol, 123.1 mg), formic acid, and additives in the given ratio were milled in a horizontal vibratory mill in a 15 mL ZrO_2_ milling jar with one milling ball (Ø = 8 mm, *m* = 3.2 g) of the same material for 200 minutes at the frequency of 30 Hz. ^c^Determined by ^1^H NMR analysis. ^d^20 milling balls (Ø = 3.0 mm, *m*_tot_ = 6.5 g) were used.

When the amine **1** was reacted in the presence of Et_3_N, HCOOH, and *p*-Ts-Im [58] (Table 1, entry 5), the formamide was accompanied by a significant amount of sulfonamide (formamide/sulfonamide ratio: 70:30). Better results were observed when *p*-Ts-Im was used under acidic conditions (see Table 1, entries 6 and 7). A slight enhancement in yields was observed when 20 balls (Ø = 3 mm, *m*_tot_ = 6.5 g) were used instead of one ball.

Recent studies on the effect of the size and number of milling balls pointed out that milling balls of larger diameter led to more rapid transformations [59–60]. On the other side, previously reported procedures revealed a beneficial effect on the reaction rate when the volume fraction occupied by balls inside the reactor increases. In addition, it was found that in some certain transformations, small milling balls are beneficial for the reaction rates since the number of stress events is significantly increased [61–62]. The stress energy appears to be less critical in these processes, and we cannot rule out a thermochemical process [63].

Furthermore, we observed that the reaction efficiently took place even in the presence of a catalytic amount of *p*-Ts-Im without significant differences in the reactivity.

This data led us to question the effective role of *p*-Ts-Im in promoting the formylation reaction, which could exploit its role as the sole solid auxiliary of grinding. *p*-Tosylimidazole releases imidazole as a byproduct during the process. Therefore, we wondered whether imidazole could promote the formation of the target formamide **2**. Imidazole, compared to *p*-tosylimidazole, is a cheaper reagent and makes the final purification process easier.

As a matter of fact, by reacting 1.0 mmol of the model substrate with 2.0 mmol of formic acid and 1.0 mmol of imidazole [23], the product was obtained in better yield and with a higher degree of purity. A control experiment performed by reacting *p*-methoxyaniline (1.0 mmol) and formic acid (2.0 mmol) provided lower conversion into the desired formamide **2** (71% NMR yield, Table S1 in Supporting Information File 1), denoting the input given by imidazole as a promoter of formamide synthesis. Further variation in the ratio of imidazole/formic acid/amine decreases the reaction yield (Table S1 in Supporting Information File 1). These data pointed out the importance of working in the presence of an excess of acid and a stoichiometric amount of imidazole.

**Scheme 1:**
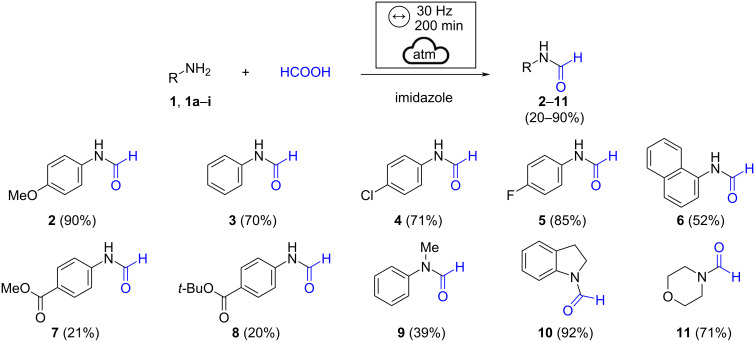
Scope of the formylation reaction using imidazole. Reaction conditions: amine (1.0 mmol), formic acid (2.0 mmol), and imidazole (1.0 mmol) were milled in a horizontal vibratory mill in a 15 mL ZrO_2_ milling jar with 20 milling balls (Ø = 3 mm, *m*_tot_ = 6.5 g) of the same material for 200 minutes at the frequency of 30 Hz.

The better results recorded in the presence of imidazole may be due to different factors. It is known that imidazole can efficiently promote those processes involving proton transfer under mechanochemical conditions. In the solvent-based procedure, imidazole has already been used in combination with DMF, which works as a solvent and source of a formyl group [23]. In that paper, the authors assumed the formation of formylimidazole as an intermediate in the formylation reaction. However, the intermediate was not detected by NMR or GC–MS analysis, possibly due to its instability [64]. Several studies carried out on our reaction crude at different times did not show the presence of compounds traceable to formylimidazole. The imidazole plays a dual role as promoting reagent and solid grinding additive and can be easily removed by aqueous acid workup. The adoption of imidazole as a grinding additive is required to avoid the slurry obtained by mixing amine and acid while allowing the formation of a waxy solid, which is more suitable for a mechanochemical action. With the optimized conditions in hand, the methodology was successfully applied to the synthesis of several formamides starting from a series of aromatic amines (Scheme 1).

The *N*-formyl derivative **3** was prepared in good yields as well as formanilides bearing halogen substituents, which were well tolerated (Scheme 1, products **4** and **5**). *N*-(1-Naphthyl)formamide (**6**) was obtained in satisfying yield, while the methyl and *tert-*butyl ester moieties affected the reaction outcome (Scheme 1, amides **7** and **8**). Secondary amines were also tested under the developed conditions; *N*-methylaniline provided the desired product **9** in 39% yield, while outstanding results were achieved in the *N*-formylation of indoline (Scheme 1, formamide **10**). The present methodology could also be effectively applied to the synthesis of *N*-formylmorpholine (Scheme 1, product **11**).

Aliphatic primary amines can be more challenging substrates [22]. In fact, when the reaction was tested with phenylethylamine (**12**), we observed only 10% of conversion into the desired formamide (Table 2, entry 1). We, therefore, chose to resume the use of *p*-Ts-Im, envisioning it could contribute to improving the reactivity. At this point, we reacted the amine **12** (1.0 mmol) with *p*-Ts-Im (1.0 mmol), and formic acid (1.0 or 2.0 mmol, Table 2, compare entries 2 and 3) for 200 minutes at the frequency of 30 Hz and observed a significant improvement on the performance of the reaction. To our delight, the complete conversion into the desired formamide **13** was obtained using 1.5 equivalents of *p*-Ts-Im. Under these experimental conditions, we did not detect the formation of the sulfonamide derivative, preserving complete selectivity towards the target formamide (Table 2, entry 4).

**Table 2:** Optimization of reaction conditions for product **13**.^a^

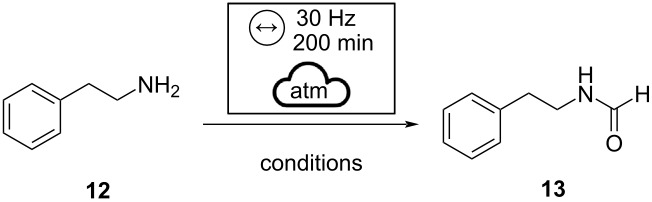			

Entry	Formic acid (equiv)	Additives (equiv)	Yield of **13** (%)^b^

1	2	imidazole (1.0)	10
2	2	*p*-Ts-Im (1.0)	42
3	1	*p*-Ts-Im (1.0)	42
4	1.5	*p*-Ts-Im (1.5)	99
**5** **^c^**	**1.5**	***p*** **-Ts-Im (1.5)**	**95**

^a^Amine **12** (1.0 mmol, 121.2 mg), formic acid, and additives in the given ratio were milled in a horizontal vibratory mill in a 15 mL ZrO_2_ milling jar with 20 milling balls (Ø = 3 mm, *m*_tot_ = 6.5 g) of the same material for 200 minutes at the frequency of 30 Hz; ^b^determined by ^1^H NMR analysis; ^c^the mixture was ball-milled for 120 minutes.

**Scheme 2:**
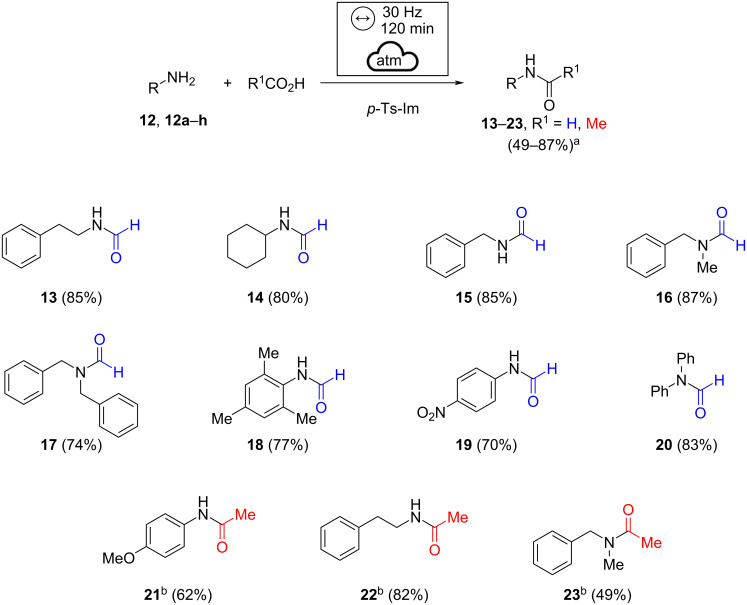
Scope of the formylation reaction using *p*-Ts-Im as activating agent. Reaction conditions: amine (1.0 mmol), formic acid (1.5 mmol), and *p*-Ts-Im (1.5 mmol) were milled in a horizontal vibratory mill in a 15 mL ZrO_2_ milling jar with 20 milling balls (Ø = 3 mm, *m*_tot_ = 6.5 g) of the same material for 120 minutes at the frequency of 30 Hz. ^a^Isolated yields. ^b^Acetic acid and *p*-Ts-Im were ball-milled for 10 minutes, then the amine was added, and the mixture was ball-milled for additional 120 minutes.

Remarkably, the results remain unchanged regarding yields and purity by shortening the reaction time (Table 2, entry 5). Furthermore, these optimized conditions were applied to other amines that provided us with poor results using the methodology previously described (Scheme 2).

We obtained the formation of formamides **14** and **15** from cyclohexylamine and benzylamine in high yields (Scheme 2). Excellent results were also achieved using secondary aliphatic amines such as dibenzyl- and methylbenzylamine, which provided amides **16** and **17** in 87% and 74% isolated yields, respectively. Furthermore, the methodology allowed us to synthesize a set of aromatic formanilides (amides **18**–**20**, Scheme 2). Indeed, the deactivated *p*-nitroaniline was successfully converted into the corresponding formamide **19** as was the poorly nucleophilic diphenylamine (Scheme 2, amide **20**).

Lastly, we aimed to apply the procedure to the acylation of a series of amines. To our delight, we successfully extended the methodology to the mechanosynthesis of amides **21**–**23** (Scheme 2). The best results were obtained when the acetic acid and the *p*-Ts-Im were ball-milled together before adding the suitable amine. In this way, we could acylate primary aromatic and aliphatic amines under the experimental conditions (Scheme 2, amides **21**–**23**).

## Conclusion

In conclusion, we developed two easily accessible ways to obtain a good number of formylated and acylated amines under mechanochemical conditions. The two methodologies exhibit complementary features as they enable the derivatization of different kinds of amines. Imidazole was found a suitable additive for the *N*-formylation reaction of several aromatic amines. On the other side, *p*-Ts-Im, activated by the acid reagent, proved an efficient activating agent under mild mechanochemical conditions for the formylation and acylation of those amines with less marked or limited (see protonated aliphatic amines) reactivity.

## Supporting Information

File 1 Experimental section and characterization of synthesized compounds.
