# Supplementary material for: From amines to (form)amides: a simple and successful mechanochemical approach
Source: Beilstein J Org Chem. 2022 Sep 12;18:1210–6. doi: 10.3762/bjoc.18.126 (PMC9490066; doi:10.3762/bjoc.18.126)

BEILSTEIN JOURNAL OF ORGANIC CHEMISTRY

Supporting Information

for

From amines to (form)amides: a simple and successful mechanochemical approach

Federico Casti, Rita Mocci and Andrea Porcheddu

Beilstein J. Org. Chem. **2022,** *18,* 1210–1216. doi:10.3762/bjoc.18.126

Experimental section and characterization of synthesized compounds

License and Terms: This is a supporting information file under the terms of the Creative Commons Attribution License (https://creativecommons.org/licenses/by/4.0). Please note that the reuse, redistribution and reproduction in particular requires that the author(s) and source are credited and that individual graphics may be subject to special legal provisions.
The license is subject to the *Beilstein Journal of Organic Chemistry* terms and conditions: (https://www.beilstein-journals.org/bjoc/terms)

Table of contents

General methods and materials S2
General experimental procedures for the synthesis of amides S3
General experimental procedure for the preparation of amides **2**–**11** S3
General experimental procedure for the preparation of amides **13**–**20** S3
General experimental procedure for the preparation of amides **21**–**23** S3
Optimization of the reaction conditions S4
Table S1: Optimization of the reaction conditions for **2** and **13**[a] S4
Spectral data for amides **2**–**11** and **13**–**22** S5
References S9
Representative GC–MS traces for compounds **2**, **18**, **20**, and **22** S10
^{1}H and ^{13}C NMR spectra for compounds **2**–**11** and **13**–**22** S12

General methods and materials

Commercially available reagents were purchased from Acros, Aldrich, Strem Chemicals, Alfa-Aesar, and TCI Europe and were used as received. All reactions were monitored by thin-layer chromatography (TLC) performed on glass-backed silica gel 60 F254, 0.2 mm plates (Merck), and compounds were visualized under UV light (254 nm) or using cerium ammonium molybdate solution with subsequent heating. The eluents were technical grade. Mechanochemical reactions were carried out using a FormTech FTS-1000 Shaker Mill® apparatus (horizontal vibratory mill). The reagents were milled using a zirconia SmartSnap™ grinding jar (15 mL) equipped with balls (Ø = 8 mm or Ø = 3 mm) of the same material. Precisely, the zirconium oxide of the vessels and balls used for all reactions accomplished in the mixer mill is yttrium oxide stabilized (ZrO_2-Y). If not stated otherwise, these parameters were applied. ^1H and ^{13}C liquid NMR spectra were recorded on a Bruker Avance III HD 600 MHz NMR spectrometer at 298 K. Proton chemical shifts are expressed in parts per million (ppm, δ scale) and are referred to the residual hydrogen of the solvent ($CDCl_3$, 7.27 ppm or DMSO 2.54 ppm) or to internal tetramethylsilane (TMS). Data are represented as follows: chemical shift δ is expressed in ppm, multiplicity (s = singlet, d = doublet, t = triplet, q = quartet, m = multiplet and/or multiple resonances, br = broad singlet, and combination of thereof), coupling constant (*J*) in hertz (Hz) and integration. Carbon chemical shifts are expressed in parts per million (ppm, δ scale) and are referenced to the carbon resonances of the NMR solvent ($CDCl_3$, δ 77.0 ppm or DMSO-d_6 δ 39.5 ppm). Deuterated NMR solvents were obtained from Aldrich. Samples were analyzed using an Agilent 5977B MS interfaced to the GC 7890B equipped with a DB-5ms column (J & W), injector temperature at 230 °C, detector temperature at 280 °C, helium carrier gas flow rate of 1 mL/min. The GC oven temperature program was 100 °C initial temperature with 4 min hold time and ramping at 15 °C/min to a final temperature of 270 °C with 7 min hold time. One µL of each sample was injected in split (1:20) mode. After a solvent delay of 3 minutes, mass spectra were acquired in full scan mode using 2.28 scans/s with a 50–500 amu mass range. Retention times of different compounds were determined by injecting pure compounds under identical conditions. HRMS were recorded on LTQ Orbitrap Elite (Thermofischer) instrument (ESI). All the experiments were carried out in duplicate to ensure the reproducibility of the experimental data. Yields refer to pure isolated materials.

General experimental procedures for the synthesis of amides

General experimental procedure for the preparation of amides 2–11

Formic acid (2.0 mmol, 75 µL), imidazole (1.0 mmol, 68.1 mg), amines **1** or **1a**–**i** (1.0 mmol) were loaded into a zirconium dioxide grinding jar (15 mL) equipped with 20 balls (Ø = 3.0 mm, m_{tot} 6.5 g) of the same material. The jar was sealed and the mechanochemical reaction was conducted for 200 min at a frequency of 30 Hz in an vibrating ball mill. Upon completion of the ball-milling process, the content of the jar was dissolved in ethyl acetate and washed with 30 mL of 2 M HCl aqueous solution (2 × 15 mL). The organic phases were filtered through a short silica plug and dried *in vacuo* to give the corresponding formamide. When necessary, further purification can be achieved by column chromatography (DCM/MeOH 98:2 v/v).

General experimental procedure for the preparation of amides 13–20

Formic acid (1.5 mmol, 57 µL), 1-(*p*-toluenesulfonyl)imidazole (1.5 mmol, 333.4 mg), and amines **12** and **12a**–**h** (1 .0 mmol) were loaded into a zirconium dioxide grinding jar (15 mL) equipped with 20 balls (Ø = 3.0 mm, m_{tot} 6.5 g) of the same material. The jar was sealed and the mechanochemical reaction was conducted for 120 min at a frequency of 30 Hz in a vibrating ball mill. Upon completion of the ball-milling process, the content of the jar was dissolved in ethyl acetate and washed with 30 mL of 2M HCl aqueous solution (2 × 15 mL). The organic phase was filtered through a short silica plug and dried *in vacuo* to give the corresponding formamide. When necessary, further purification can be achieved by column chromatography (DCM/MeOH 98:2 v/v).

General experimental procedure for the preparation of amides 21–23

Acetic acid (1.5 mmol, 114 µL), 1-(*p*-toluenesulfonyl)imidazole (1.5 mmol, 333.4 mg), amine **1** or **12** (1.0 mmol) were loaded into a zirconium dioxide grinding jar (15 mL) equipped with 20 balls (Ø = 3.0 mm, m_{tot} 6.5 g) of the same material. The jar was sealed and the mechanochemical reaction was conducted for 120 min at a frequency of 30 Hz in a vibrating ball mill. Upon completion of the ball-milling process, the content of the jar was dissolved in ethyl acetate and washed with 30 mL of 2M HCl aqueous solution (2 × 15 mL). The organic phase was filtered through a short silica plug and dried *in vacuo* to give the corresponding formamide. When necessary, further purification can be achieved by column chromatography (DCM/MeOH 98:2 v/v).

Optimization of the reaction conditions

Table S1: Optimization of the reaction conditions for 2 and 13[a,b]

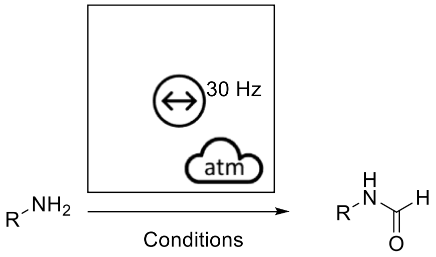

Entry[c]	R	Formic acid (equiv)	Additives (equiv)	Formamide (%)
1	*p*-(OMe)Ar-	1.0	–	59
2	*p*-(OMe)Ar-	1.5	–	65
3	*p*-(OMe)Ar-	2.0	–	71
4	*p*-(OMe)Ar-	1.0	imidazole (1.0)	44
5	*p*-(OMe)Ar-	2.0	imidazole (2.0)	55
6	$PhCH_2CH_2$-	1.5	Na_2SO_4 (2.8)	–
7	$PhCH_2CH_2$-	1.5	Na_2SO_4 (2.8)/DMU (1.0)	–
8	$PhCH_2CH_2$-	1.5	Na_2SO_4 (2.8)/imidazole (1.0)	5
9	$PhCH_2CH_2$-	1.5	*p*-Ts-Im (10 mol %)	10

[a]The reaction scheme was depicted using the symbolism proposed in [1]. [b]Conditions: **1** (1.0 mmol, 123.1 mg), formic acid, and additives in the given ratio were milled in a vibratory mill in a ZrO_2 milling jar (15 mL) with 20 milling balls (Ø = 3.0 mm, m_{tot} = 6.5 g) for 200 minutes at the frequency of 30 Hz. [c]Determined by ^1H NMR analysis.

Spectral data for amides 2–11 and 13–22

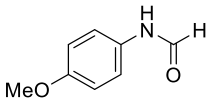

***N*-(4-Methoxyphenyl)formamide (2).** Pale brownish solid; 136 mg, 90% yield. The product was obtained in the form of 2 rotamers. **^{1}H NMR** (600 MHz, $CDCl_3$) δ 8.53 (br s, 0.39H) 8.50 (d, *J* = 11.2 Hz, 0.56H), 8.27 (s, 0.53H), 7.90 (br s, 0.47H), 7.43 (d, *J* = 8.9 Hz, 1H), 7.02 (d, *J* = 8.9 Hz, 1H), 6.85 (dd, *J* = 22.2, 8.9 Hz, 2H), 3.78 (s, 1.45H), 3.76 (s, 1.55H). **^{13}C NMR** (151 MHz, $CDCl_3$) δ 163.4, 159.3, 157.6, 156.7, 130.2, 129.7, 121.9, 121.6, 115.0, 114.3, 55.6, 55.5. All spectral data are consistent with previously published findings.[2]

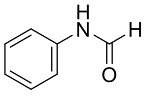

***N*-Phenylformamide (3).** Orange oil, 85 mg, 70% yield. The product was obtained in the form of 2 rotamers.**^{1}H NMR** (600 MHz, $CDCl_3$) δ 8.69 (d, *J* = 11.4 Hz, 0.55H), 8.39 (s, 0.45H), 8.02 (br s, 0.51H), 7.58 – 7.51 (m, 1H), 7.39 – 7.32 (m, 2H), 7.30 (br s, 0.48H), 7.23 – 7.19 (m, 0.59H), 7.17 – 7.13 (m, 0.50H), 7.13 – 7.06 (m, 1H). **^{13}C NMR** (151 MHz, $CDCl_3$) δ 162.6, 159.0, 136.9, 136.7, 129.9, 129.3, 125.5, 125.0, 120.1, 119.0. All spectral data are consistent with previously published findings.[3]

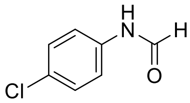

***N*-(4-Chlorophenyl)formamide (4).** Pale brownish solid; 110 mg, 71% yield. The product was obtained in the form of 2 rotamers. **^{1}H NMR** (600 MHz, $CDCl_3$) δ 8.66 (br s, 0.43H), 8.64 (s, 0.39H), 8.35 (s, 0.58H), 7.71 (br s, 0.54H), 7.49 (d, *J* = 8.5 Hz, 1H), 7.37 – 7.25 (m, 2H), 7.04 (d, *J* = 8.5 Hz, 0.83H). **^{13}C NMR** (151 MHz, $CDCl_3$) δ 162.7, 159.2, 135.5, 135.4, 130.9, 130.0, 130.0, 129.2, 121.4, 120.2.
All spectral data are consistent with previously published findings.[4]

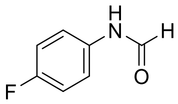

***N*-(4-Fluorophenyl)formamide (5).** Pale brownish solid; 118 mg, 85% yield. **^{1}H NMR** (600 MHz, $CDCl_3$) δ 8.57 (d, *J* = 11.3 Hz, 0.45H), 8.50 (br s, 0.41H), 8.34 (d, *J* = 1.8 Hz, 0.55H), 7.64 (br s, 0.59H) 7.53 – 7.47 (m, 1.18), 7.10 – 7.04 (m, 1.66H), 7.01 (t, *J* = 8.6 Hz, 1.18H). **^{13}C NMR** (151 MHz, $CDCl_3$) δ 163.1, 160.2 (d, *J* = 244,6 Hz), 159.7 (d, *J* = 244,6 Hz), 159.2, (151 MHz, $CDCl_3$) 133.0 (d, *J* = 2.9 Hz), 132.8 (d, *J* = 3.2 Hz), 122.0 (d, *J* = 7.5 Hz), 121.3 (d, *J* = 9.1 Hz), 116.7 (d, *J* = 23.1 Hz), 115.9 (d, *J* = 22.4 Hz). All spectral data are consistent with previously published findings.[4]

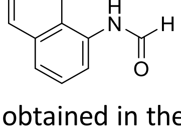

***N*-(Naphthalen-1-yl)formamide (6)** Pale brownish solid; 89 mg, 52% yield. The product was obtained in the form of 2 rotamers. **^{1}H NMR** (600 MHz, $CDCl_3$) δ 8.63 (d, *J* = 7.5 Hz, 0.59H), 8.62 (s, 0.49H), 8.51 (br s, 0.65H), 8.01 (t, *J* = 7.9 Hz, 1.03H), 7.92 – 7.89 (m, 0.83H), 7.87 (d, *J* = 8.0 Hz, 0.57H), 7.80 (d, *J* = 8.3 Hz, 0.72H), 7.70 (br s, 0.35H), 7.62 – 7.47 (q, *J* = 7.7 Hz, 3.06H), 7.32 (d, *J* = 7.3 Hz, 0.72H). **^{13}C NMR** (151 MHz, $CDCl_3$) δ 164.2, 159.7, 134.4, 134.2, 132.2, 131.1, 129.0, 128.7, 127.9, 127.2, 127.2, 127.0, 126.7, 126.8, 126.4, 126.3, 125.8, 125.6, 121.4, 121.0, 120.5, 119.2. All spectral data are consistent with previously published findings.[5]

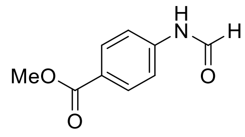

Methyl 4-formamidobenzoate (7). White solid; 38 mg, 21% yield. **^{1}H NMR** (600 MHz, $CDCl_3$) δ 8.85 (d, *J* = 11.2 Hz, 0.43H), 8.53 (br s, 0.39H), 8.43 (s, *J* = 1.7 Hz, 0.57H), 8.04-8.00 (m, 2H), 7.71 (br s, 0.46H), 7.63 (d, *J* = 8.5 Hz, 1.18H), 7.14 (d, *J* = 8.4 Hz, 0.91H), 3.91 (s, 1.46H), 3.90 (s, 1.54H). **^{13}C NMR** (151 MHz, $CDCl_3$) δ 166.6, 166.4, 162.1, 159.2, 141.1, 141.0, 131.7, 131.0, 126.8, 126.3, 119.2, 117.3, 52.3, 52.2. All spectral data are consistent with previously published findings.[4]

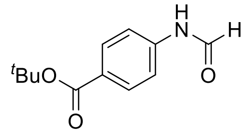

***tert*-Butyl 4-formamidobenzoate (8)** Brown oil; 46 mg, 20% yield. **^{1}H NMR** (600 MHz, $CDCl_3$) δ 8.82 (d, *J* = 11.1 Hz, 0.47H), 8.66 (br s, 0.43H), 8.42 (s, 0.56H), 7.98-7.94 (m 2H), 7.85 (br s, 0.49H), 7.61 (d, *J* = 8.5 Hz, 1.06H), 7.11 (d, *J* = 8.4 Hz, 1H), 1.59 (s, 3.75H), 1.58 (5.25H). **^{13}C NMR** (151 MHz, $CDCl_3$) δ 165.4, 165.1, 162.4, 159.3, 140.7, 140.6, 131.5, 131.5, 130.8, 128.7, 128.2, 119.1, 117.3, 81.4, 81.2, 28.3.

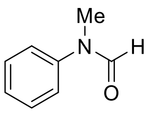

***N*-Methyl-*N*-phenylformamide (9).** Yellow oil; 53 mg, 39% yield. **^{1}H NMR** (600 MHz, $CDCl_3$) δ 8.47 (s, 1H), 7.41 (t, *J* = 7.8 Hz, 2H), 7.28 (t, *J* = 7.4 Hz, 1H), 7.20 – 7.15 (m, 2H), 3.32 (s, 3H). **^{13}C NMR** (151 MHz, $CDCl_3$) δ 162.6, 142.3, 129.8, 126.6, 122.6, 32.2. All spectral data are consistent with previously published findings.[6]

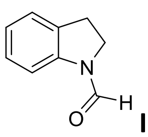

Indoline-1-carbaldehyde (10). Brown oil; 135 mg, 92%. The product was obtained in the form of 2 rotamers. **^{1}H NMR** (600 MHz, $CDCl_3$) δ 8.87 (s, 0.78H), 8.46 (s, 0.18H), 8.02 (d, *J* = 7.9 Hz, 0.19H), 7.20 – 7.09 (m, 3H), 7.01 (td, *J* = 7.3, 1.3 Hz, 0.96H), 4.04 (t, *J* = 8.4 Hz, 0.42H), 3.99 (t, *J* = 8.5 Hz, 1.58H), 3.13-3.10 (m, 0.47H), 3.09 (t, *J* = 8.5 Hz, 1.53H). **^{13}C NMR** (151 MHz, $CDCl_3$) δ 159.3, 157.5, 141.2, 141.0, 132.0, 131.9, 127.5, 127.5, 126.0, 124.8, 124.5, 124.2, 116.5, 109.3, 46.9, 44.6, 27.7, 27.1. All spectral data are consistent with previously published findings.[7]

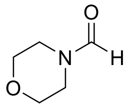

Morpholine-4-carbaldehyde (11). Colourless oil; 82 mg, 71%. The content of the jar was dissolved in ethyl acetate and purified by column chromatography (DCM:MeOH 98:2 v/v). **^{1}H NMR** (600 MHz, $CDCl_3$) δ 8.05 (s, 1H), 3.73 – 3.63 (m, 4H), 3.57 (d, *J* = 5.0 Hz, 2H), 3.39 (t, *J* = 4.8 Hz, 2H). **^{13}C NMR** (151 MHz, $CDCl_3$) δ 161.0, 67.4, 66.6, 45.9, 40.7. All spectral data are consistent with previously published findings.[8]

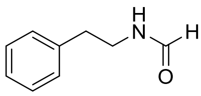

***N*-Phenethylformamide (13).** Colourless oil, 127 mg, 85% yield. The product was obtained in the form of 2 rotamers. **^{1}H NMR** (600 MHz, $CDCl_3$) δ 8.06 (s, 0.81H), 7.85 (d, *J* = 11.9 Hz, 0.17H), 7.33 – 7.27 (m, 1.95H), 7.25 – 7.15 (m, 2.97H), 5.97 (br s, 0.87H), 3.54 (q, *J* = 6.7 Hz, 1.69H), 3.44 (q, *J* = 6.8 Hz, 0.35H), 2.81 (dt, *J* = 16.5, 6.9 Hz, 2H). **^{13}C NMR** (151 MHz, $CDCl_3$) δ 164.6, 161.4, 138.6, 137.7, 128.9, 128.9, 128.9, 128.7, 126.9, 126.7, 43.2, 39.2, 37.7, 35.5. All spectral data are consistent with previously published findings.[8]

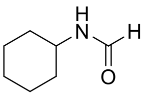

***N*-Cyclohexylformamide (14).** Colourless oil; 102 mg, 80% yield. The product was obtained in the form of 2 rotamers. **^{1}H NMR** (600 MHz, $CDCl_3$) δ 8.19 – 8.04 (m, 1H), 5.56 (br s, 0.29H), 5.40 (br s, 0.64H), 3.87 (dtd, *J* = 10.8, 7.3, 4.2 Hz, 0.68H), 3.32 (dd, *J* = 9.5, 4.7 Hz, 0.24H), 1.92 (ddt, *J* = 23.6, 12.8, 4.1 Hz, 1.81H), 1.78 – 1.59 (m, 3.99H), 1.42 – 1.31 (m, 2H), 1.12 (m, 1.21H). **^{13}C NMR** (151 MHz, $CDCl_3$) δ 163.6, 160.4, 51.0, 47.2, 34.9, 33.2, 25.6, 25.2, 24.9 (2C). All spectral data are consistent with previously published findings.[8]

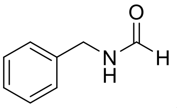

***N*-Benzylformamide (15)** Colorless crystals; 115 mg, 85% yield. The product was obtained in the form of 2 rotamers. **^{1}H NMR** (600 MHz, $CDCl_3$) δ 8.27 (s, 0.77H), 8.20 (d, *J* = 11.9 Hz, 0.15H), 7.39 – 7.23 (m, 5H), 5.83 (br s, 1H), 4.49 (d, *J* = 5.9 Hz, 1.57H), 4.42 (d, *J* = 6.5 Hz, 0.28H). **^{13}C NMR** (151 MHz, $CDCl_3$) δ 164.7, 161.0, 137.7, 137.6, 129.1, 129.0, 128.1, 128.0, 127.9, 127.1, 45.8, 42.4. All spectral data are consistent with previously published finding.[9]

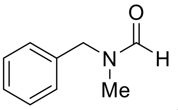

***N*-Benzyl-*N*-methylformamide (16).** White crystals, 130 mg, 87% yield. The product was obtained in the form of 2 rotamers. **^{1}H NMR** (600 MHz, $CDCl_3$) δ 8.28 (s, 0.55H), 8.15 (s, 0.41H), 7.38 – 7.26 (m, 3.34H), 7.26 – 7.22 (m, 0.89H), 7.22 – 7.17 (m, 1.13H), 4.51 (s, 0.86H), 4.38 (s, 1.15H), 2.83 (s, 1.27H), 2.77 (s, 1.67H). **^{13}C NMR** (151 MHz, $CDCl_3$) δ 162.9, 162.7, 135.9, 135.7, 128.8, 128.6, 128.2, 128.1, 127.6, 127.4, 53.5, 47.7, 34.1, 29.4. All spectral data are consistent with previously published findings.[8]

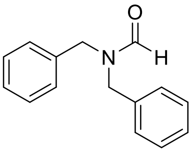

***N,N*-Dibenzylformamide (17).** White crystals, 167 mg, 74%. **^{1}H NMR** (600 MHz, $CDCl_3$) δ 8.49 (s, 1H), 7.41 – 7.27 (m, 6H), 7.24 – 7.16 (m, 4H), 4.43 (s, 2H), 4.28 (s, 2H). **^{13}C NMR** (151 MHz, $CDCl_3$) δ 163.3, 136.2, 135.8, 129.1, 128.8, 128.7, 128.3, 127.9, 127.8, 50.4, 44.8. All spectral data are consistent with previously published finding.[10]

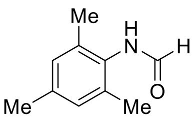

***N*-Mesitylformamide. (18).** White solid; 126 mg, 77% yield. The product was obtained in the form of 2 rotamers. **^{1}H NMR** (600 MHz, $CDCl_3$) δ 8.40 (s, 0.5H), 8.05 (d, *J* = 12.0 Hz, 0.54H), 6.93 (s, 1H), 6.91 (s, 0.94H), 6.78 (br s, 0.53H), 6.69 (br s, 0.47H) 2.29 (s, 1.53H), 2.27 (s, 1.48H), 2.26 (s, 3H), 2.22 (s, 3H). **^{13}C NMR** (151 MHz, $CDCl_3$) δ 165.0, 159.6, 137.8, 137.7, 135.4, 135.2, 129.8, 129.6, 129.5, 129.2, 21.1, 21.0, 18.8, 18.6. All spectral data are consistent with previously published findings[4]

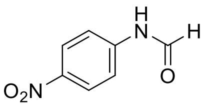

***N*-(4-Nitrophenyl)formamide (19).** Yellow solid; 116 mg, 70% yield. The product was obtained in the form of 2 rotamers. **^{1}H NMR** (600 MHz, DMSO-d_6) δ 10.80 (br s, 0.74H), 10.70 (s, br s 0.21H), 9.05 (d, *J* = 9.9 Hz, 0.24H), 8.40 (s, 0.72H), 8.23-8.19 (m, *J* = 18.6, 8.7 Hz, 2H), 7.82 (d, *J* = 8.8 Hz, 1.58H), 7.42 (d, *J* = 8.6 Hz, 0.46H). **^{13}C NMR** (151 MHz, DMSO-d_6) δ 162.8, 160.5, 144.2, 142.5, 125.5, 125.1 (2C), 119.0 (2C), 116.6. All spectral data are consistent with previously published findings.[11]

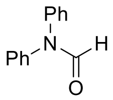

***N,N*-Diphenylformamide (20).** Pale brown solid; 164 mg, 83% yield. **^{1}H NMR** (600 MHz, $CDCl_3$) δ 8.66 (s, 1H), 7.39 (q, *J* = 7.7 Hz, 4H), 7.33 – 7.24 (m, 4H), 7.16 (dd, *J* = 7.6, 1.6 Hz, 2H). **^{1}H NMR** (600 MHz, $CDCl_3$) δ 8.66 (s, 1H), 7.39 (q, *J* = 7.7 Hz, 4H), 7.33 – 7.24 (m, 4H), 7.16 (dd, *J* = 7.6, 1.6 Hz, 2H). **^{13}C NMR (151 MHz, $CDCl_3$)** δ 161.8, 141.9, 139.7, 129.8, 129.3, 127.1, 127.0, 126.2, 125.2. All spectral data are consistent with previously published findings [3]

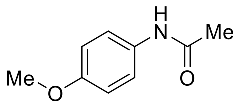

***N*-(4-Methoxyphenyl)acetamide (21).** White crystals; 102 mg, 62% yield. **^{1}H NMR** (600 MHz, DMSO-d_6) δ 9.75 (s, 1H), 7.50 – 7.41 (m, 2H), 6.89 – 6.82 (m, 2H), 3.71 (s, 3H), 1.99 (s, 3H). **^{13}C NMR** (151 MHz, DMSO-d_6) δ 167.7, 155.0, 132.5, 120.5, 113.8, 55.1, 23.8. All spectral data are consistent with previously published findings.[12]

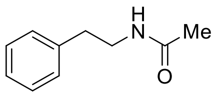

***N*-Phenethylacetamide (22).** Colourless oil; 134 mg, 82% yield. **^{1}H NMR** (600 MHz, $CDCl_3$) δ 7.30 (t, *J* = 7.5 Hz, 2H), 7.23 (d, *J* = 7.3 Hz, 1H), 7.18 (d, *J* = 7.5 Hz, 2H), 5.65 (s, 1H), 3.50 (d, *J* = 6.6 Hz, 2H), 2.81 (t, *J* = 7.0 Hz, 2H), 1.93 (s, 3H). **^{13}C NMR** (151 MHz, $CDCl_3$) δ 170.3, 139.0, 128.8, 128.7, 126.6, 40.8, 35.7, 23.4. All spectral data are consistent with previously published findings.[13]

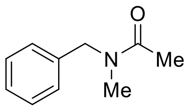

***N*-Benzyl-*N*-methylacetamide (23).** Colourless oil; 80 mg, 49% yield. The product was obtained in the form of 2 rotamers. **^{1}H NMR** (600 MHz, $CDCl_3$) δ 7.34 (t, *J* = 7.5 Hz, 1.04H), 7.32 – 7.28 (m, 1.37H), 7.26 – 7.19 (m, 1.81H), 7.15 (d, *J* = 7.6 Hz, 0.85H), 4.56 (s, 0.86H), 4.50 (s, 1.13H), 2.92 (s, 1.29H), 2.89 (s, 1.66H), 2.13 (s, 3H). **^{13}C NMR** (151 MHz, $CDCl_3$) δ 171.0, 170.7, 137.2, 136.4, 128.8, 128.4, 127.8, 127.5, 127.2, 126.2, 54.1, 50.4, 35.4, 33.5, 21.6, 21.2. All spectral data are consistent with previously published findings.[14]

References

(1) Michalchuk, A. A. L.; Boldyreva, E. V.; Belenguer, A. M.; Emmerling, F.; Boldyrev, V. V. *Front. Chem. (Lausanne, Switz.)* **2021**, *9*, 685789. doi:10.3389/fchem.2021.685789

(2) Bao, K.; Zhang, W.; Bu, X.; Song, Z.; Zhang, L.; Cheng, M. *Chem. Commun.* **2008**, No. 42, 5429. doi:10.1039/b810086a

(3) Hosseini-Sarvari, M.; Sharghi, H. *J. Org. Chem.* **2006**, *71* (17), 6652–6654. doi:10.1021/jo060847z

(4) Yin, J.; Zhang, J.; Cai, C.; Deng, G.-J.; Gong, H. *Org. Lett.* **2019**, *21* (2), 387–392. doi:10.1021/acs.orglett.8b03542

(5) Liu, H.; Nie, Z.; Shao, J.; Chen, W.; Yu, Y. *Green Chem.* **2019**, *21* (13), 3552–3555. doi:10.1039/C9GC01137D

(6) Song, R.-J.; Liu, Y.; Hu, R.-X.; Liu, Y.-Y.; Wu, J.-C.; Yang, X.-H.; Li, J.-H. *Adv. Synth. Catal.* **2011**, *353* (9), 1467–1473. doi:10.1002/adsc.201100225

(7) Kulkarni, A.; Gianatassio, R.; Török, B. *Synthesis (Stuttg).* **2011**, *2011* (08), 1227–1232. doi:10.1055/s-0030-1259978

(8) Saidi, O.; Bamford, M. J.; Blacker, A. J.; Lynch, J.; Marsden, S. P.; Plucinski, P.; Watson, R. J.; Williams, J. M. J. *Tetrahedron Lett.* **2010**, *51* (44), 5804–5806. doi:10.1016/j.tetlet.2010.08.106

(9) Suchý, M.; Elmehriki, A. A. H.; Hudson, R. H. E. *Org. Lett.* **2011**, *13* (15), 3952–3955. doi:10.1021/ol201475j

(10) Lee, H.-L.; Aubé, J. *Tetrahedron* **2007**, *63* (36), 9007–9015. doi:10.1016/j.tet.2007.05.079

(11) Fu, R.; Yang, Y.; Chen, Z.; Lai, W.; Ma, Y.; Wang, Q.; Yuan, R. *Tetrahedron* **2014**, *70* (50), 9492–9499. doi:10.1016/j.tet.2014.10.066

(12) Das Sharma, S.; Gogoi, P.; Boruah, M.; Konwar, D. *Synth. Commun.* **2007**, *37* (15), 2473–2481. doi:10.1080/00397910701459571

(13) Hanada, S.; Yuasa, A.; Kuroiwa, H.; Motoyama, Y.; Nagashima, H. *European J. Org. Chem.* **2010**, *2010* (6), 1021–1025. doi:10.1002/ejoc.200901279

(14) Nishiwaki, K.; Ogawa, T.; Shigeta, K.; Takahashi, K.; Matsuo, K. *Tetrahedron* **2006**, *62* (29), 7034–7042. doi:10.1016/j.tet.2006.04.063

Representative GC–MS traces for compounds 2, 18, 20, and 22

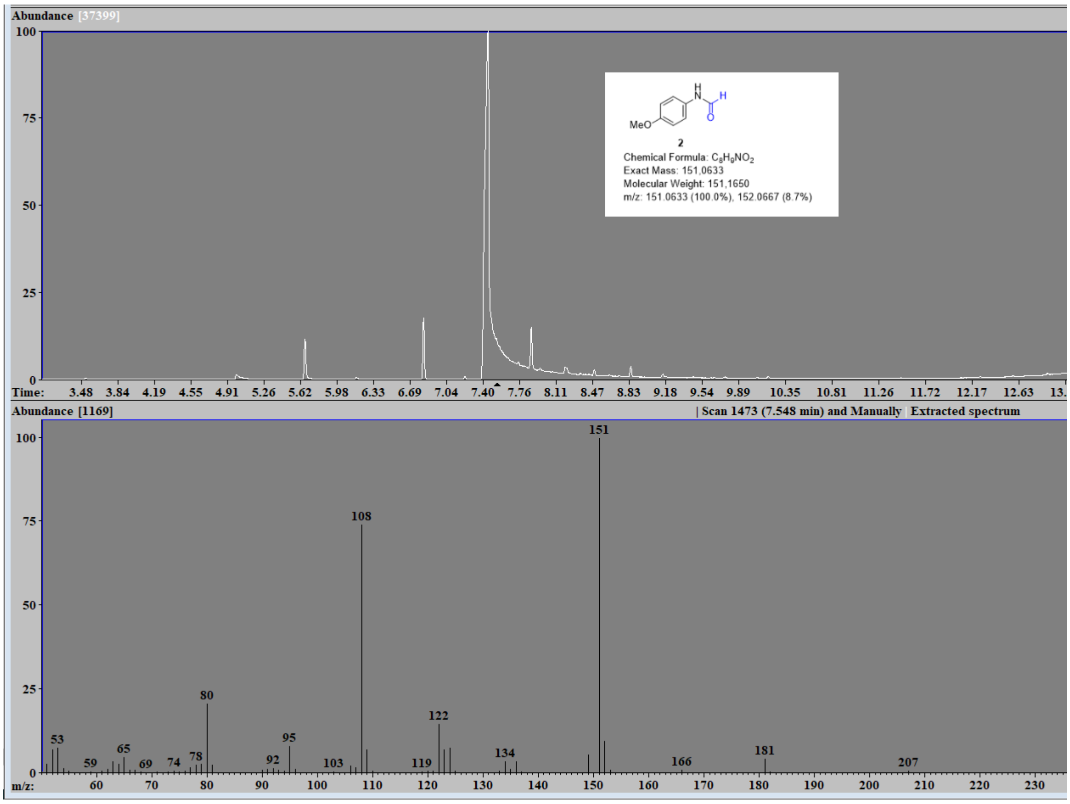

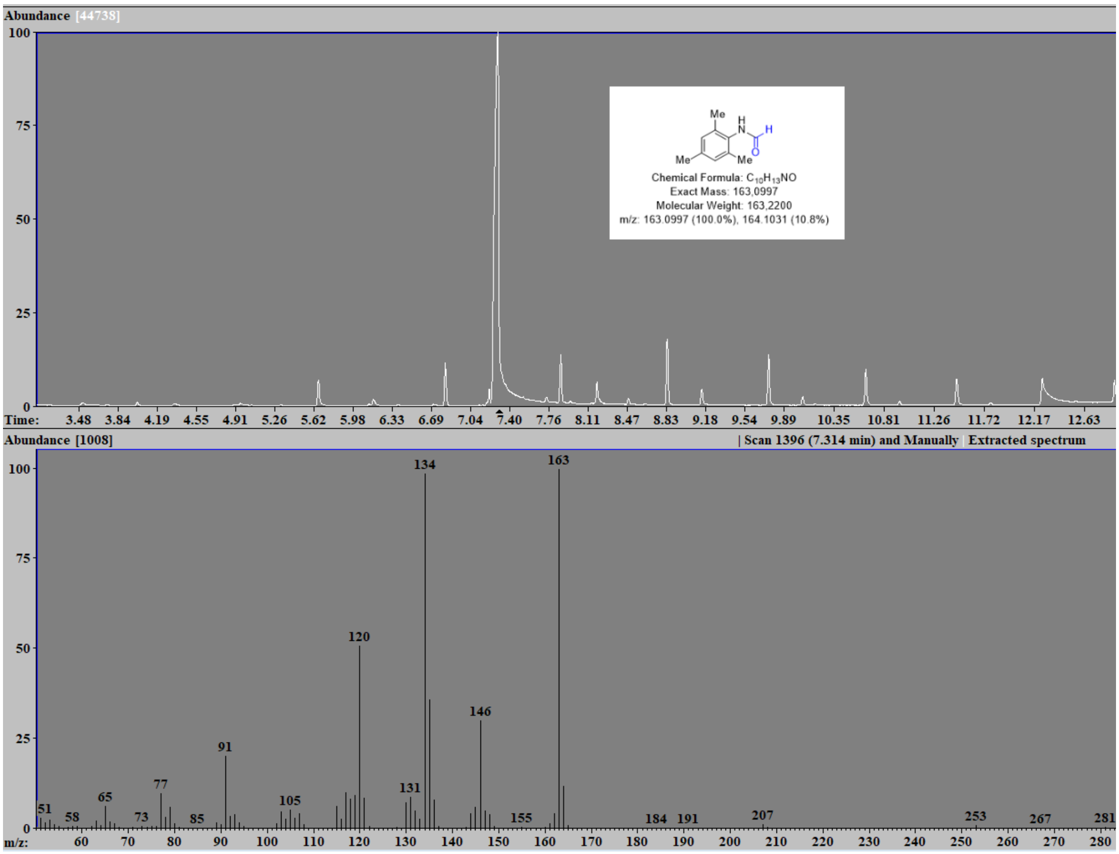

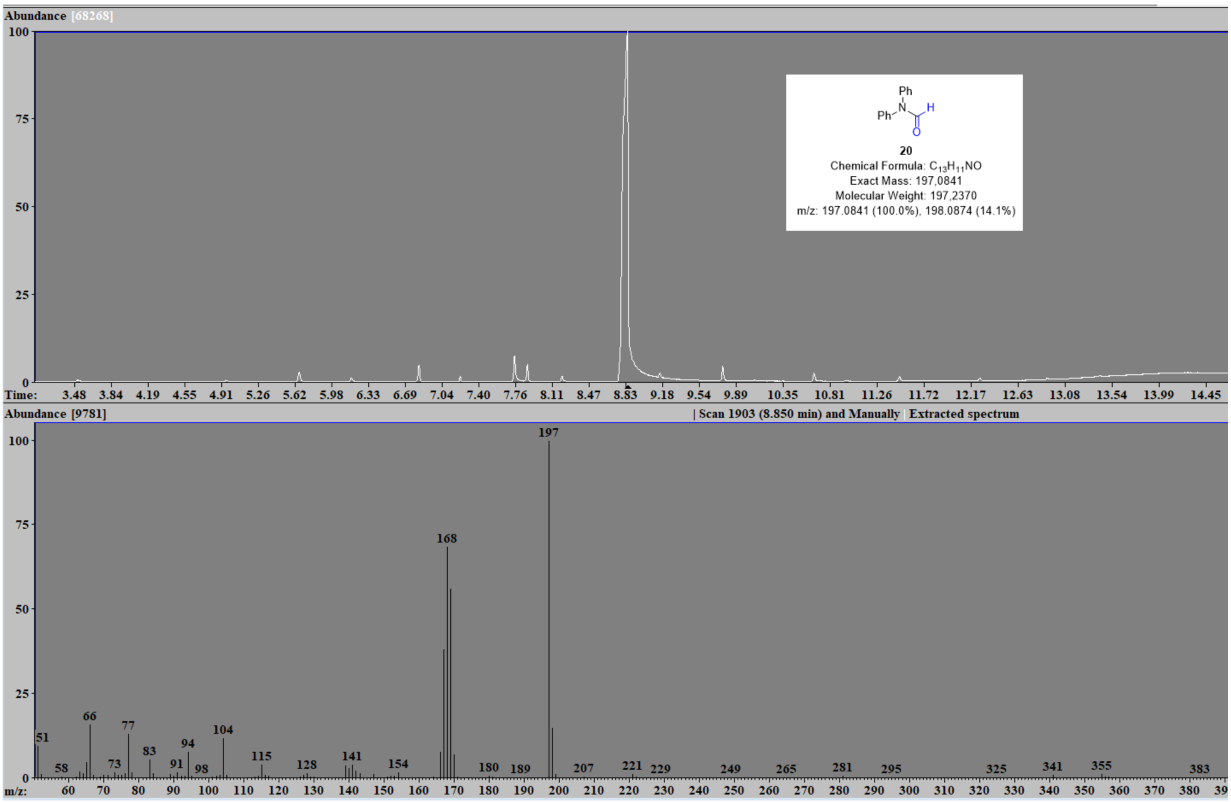
Abundance [68268]
Time:
Abundance [9781]
| Scan 1903 (8.850 min) and Manually Extracted spectrum
m/z:
20
Chemical Formula: $C_{13}H_{11}NO$
Exact Mass: 197,0841
Molecular Weight: 197,2370
m/z: 197.0841 (100.0%), 198.0874 (14.1%)

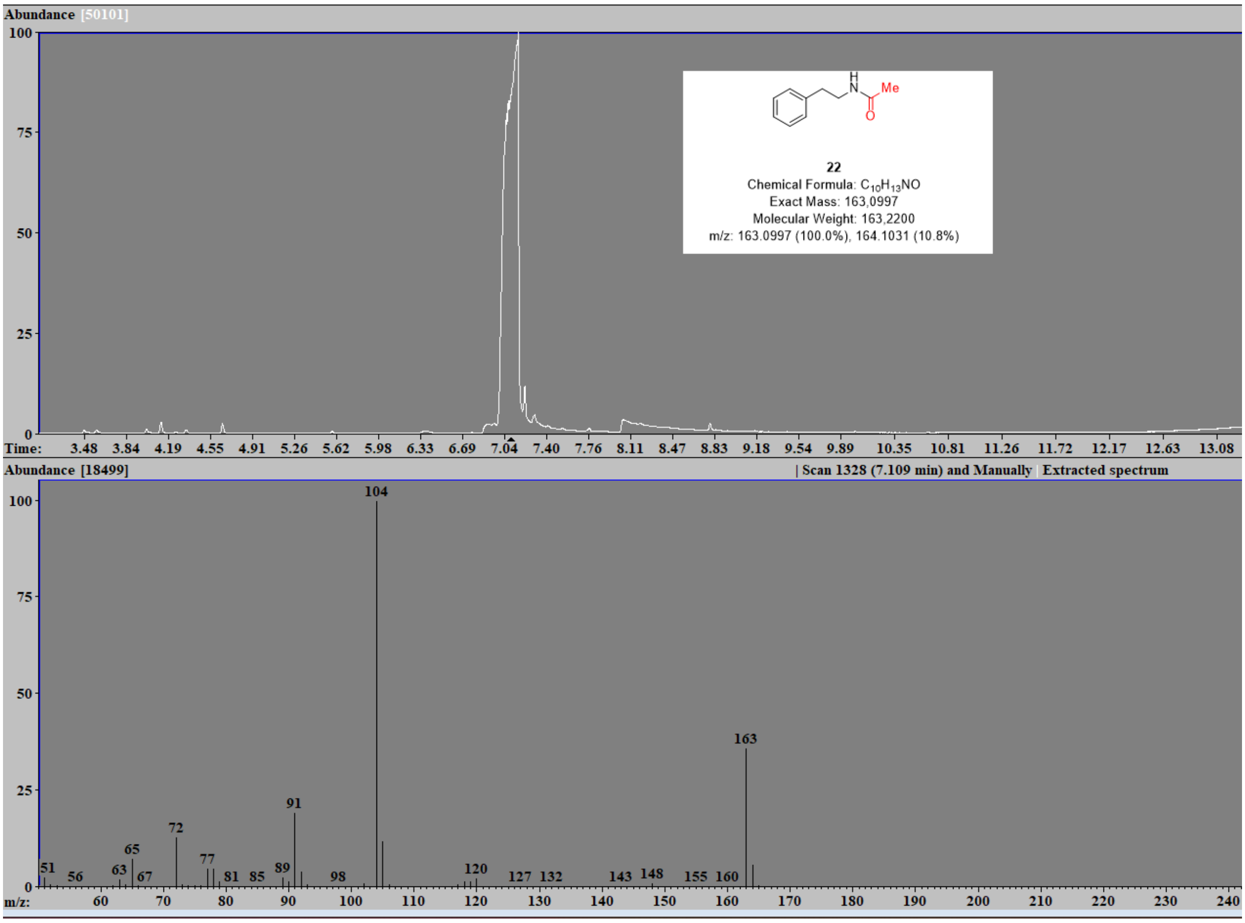
Abundance [50101]
Time:
Abundance [18499]
| Scan 1328 (7.109 min) and Manually Extracted spectrum
m/z:
22
Chemical Formula: $C_{10}H_{13}NO$
Exact Mass: 163,0997
Molecular Weight: 163,2200
m/z: 163.0997 (100.0%), 164.1031 (10.8%)

^{1}H and ^{13}C NMR spectra for compounds 2–11 and 13–22

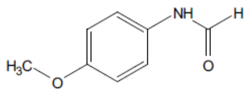

N-(4-methoxyphenyl)formamide (2)

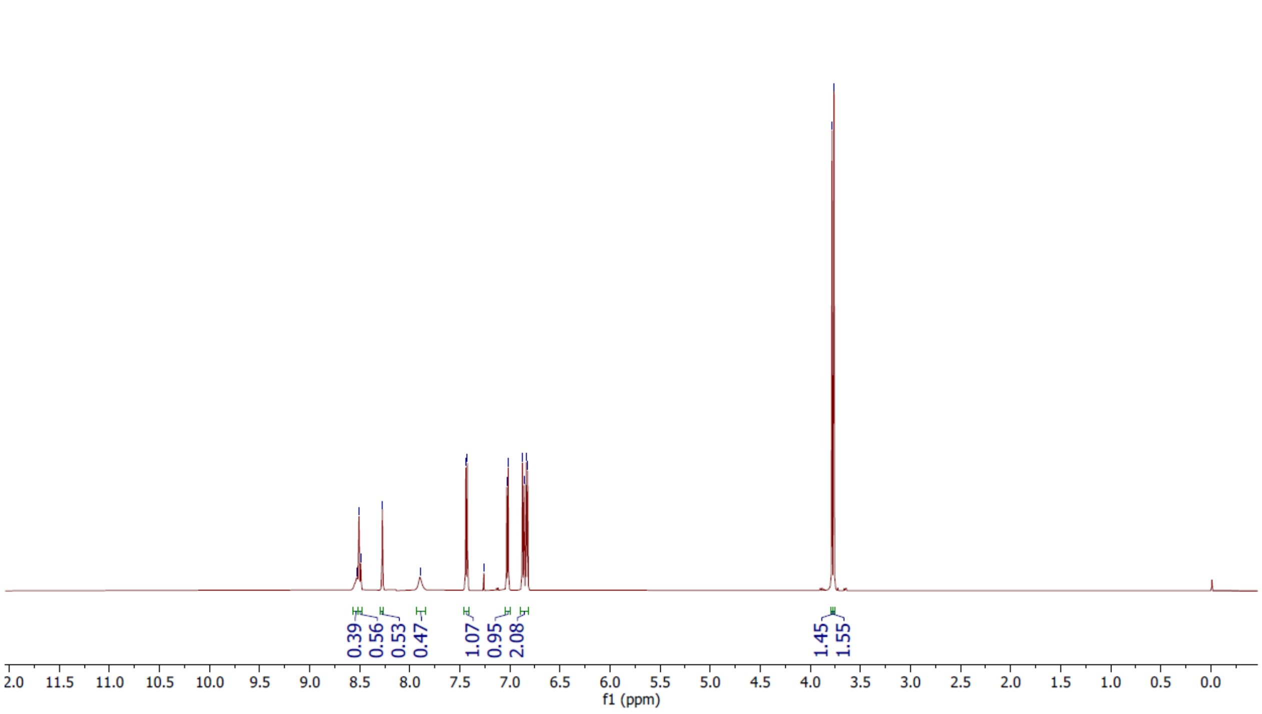

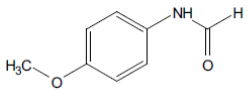

N-(4-methoxyphenyl)formamide (2)

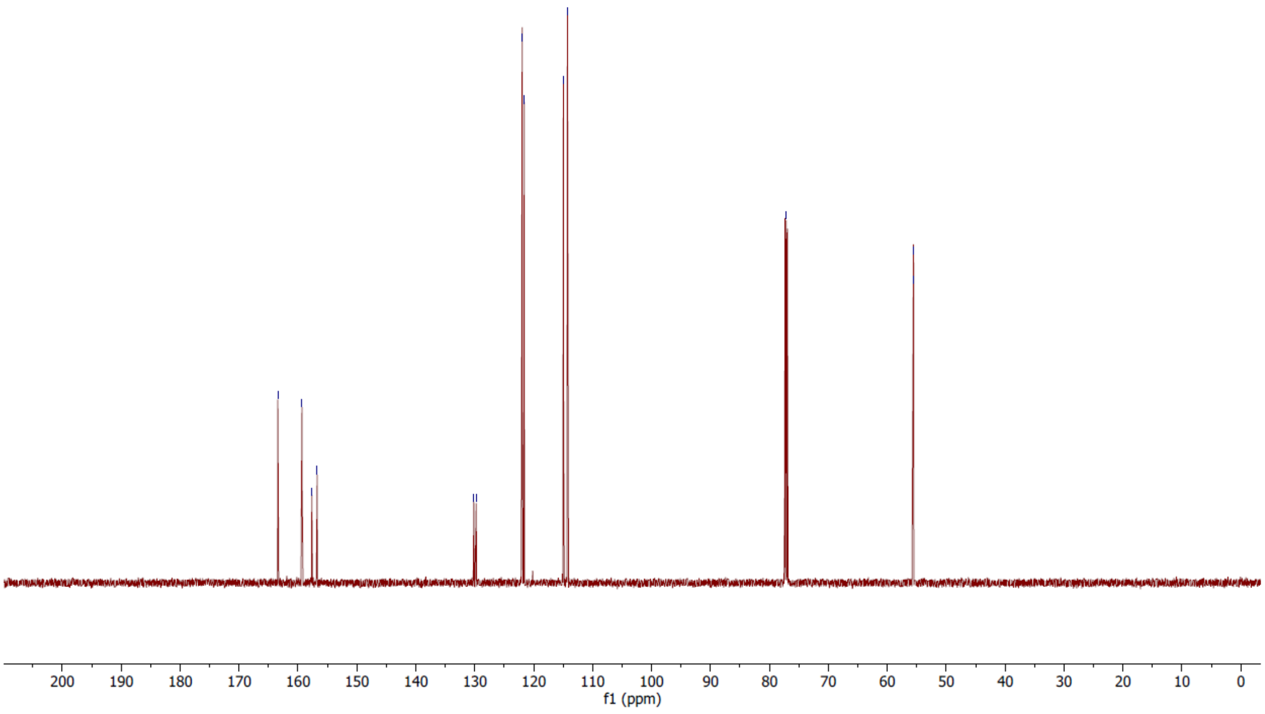

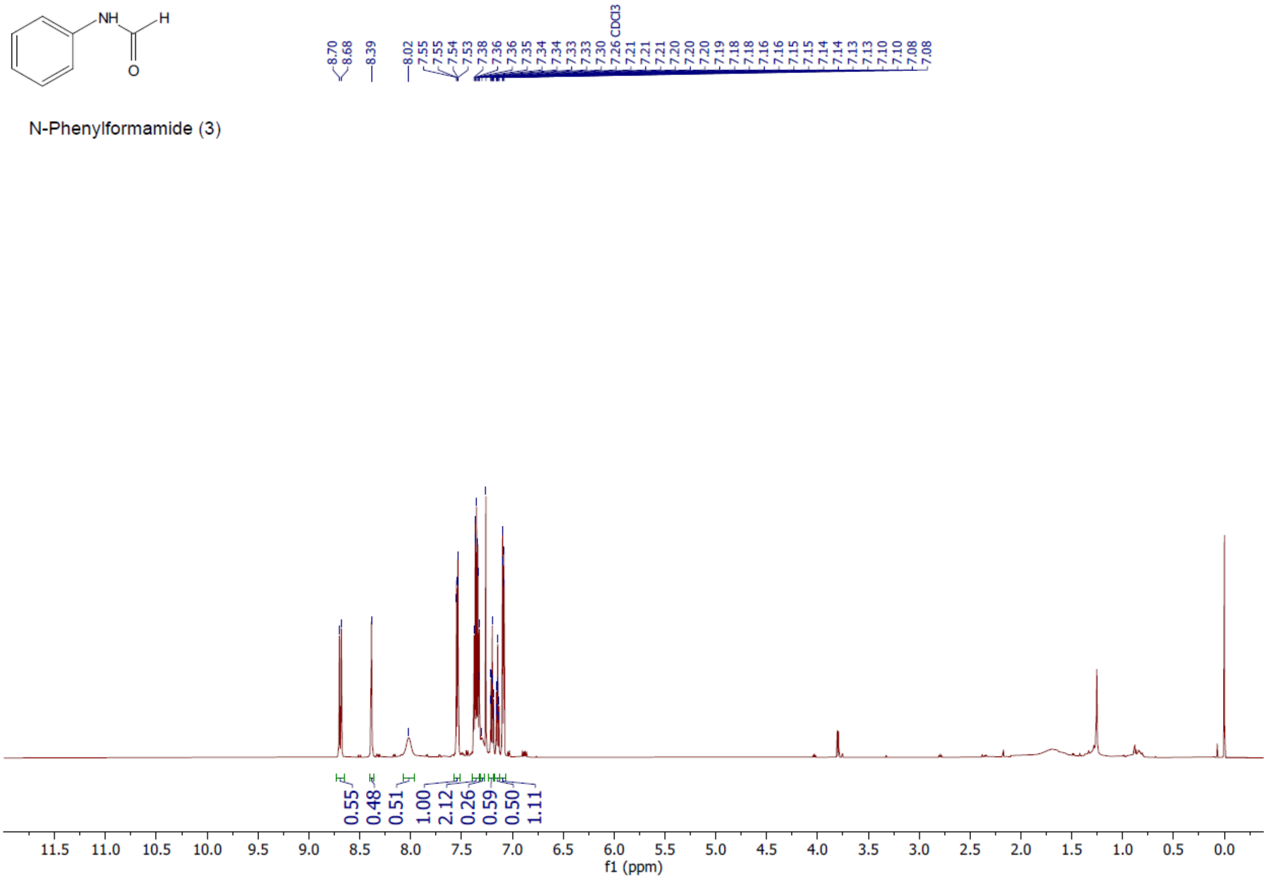

N-Phenylformamide (3)

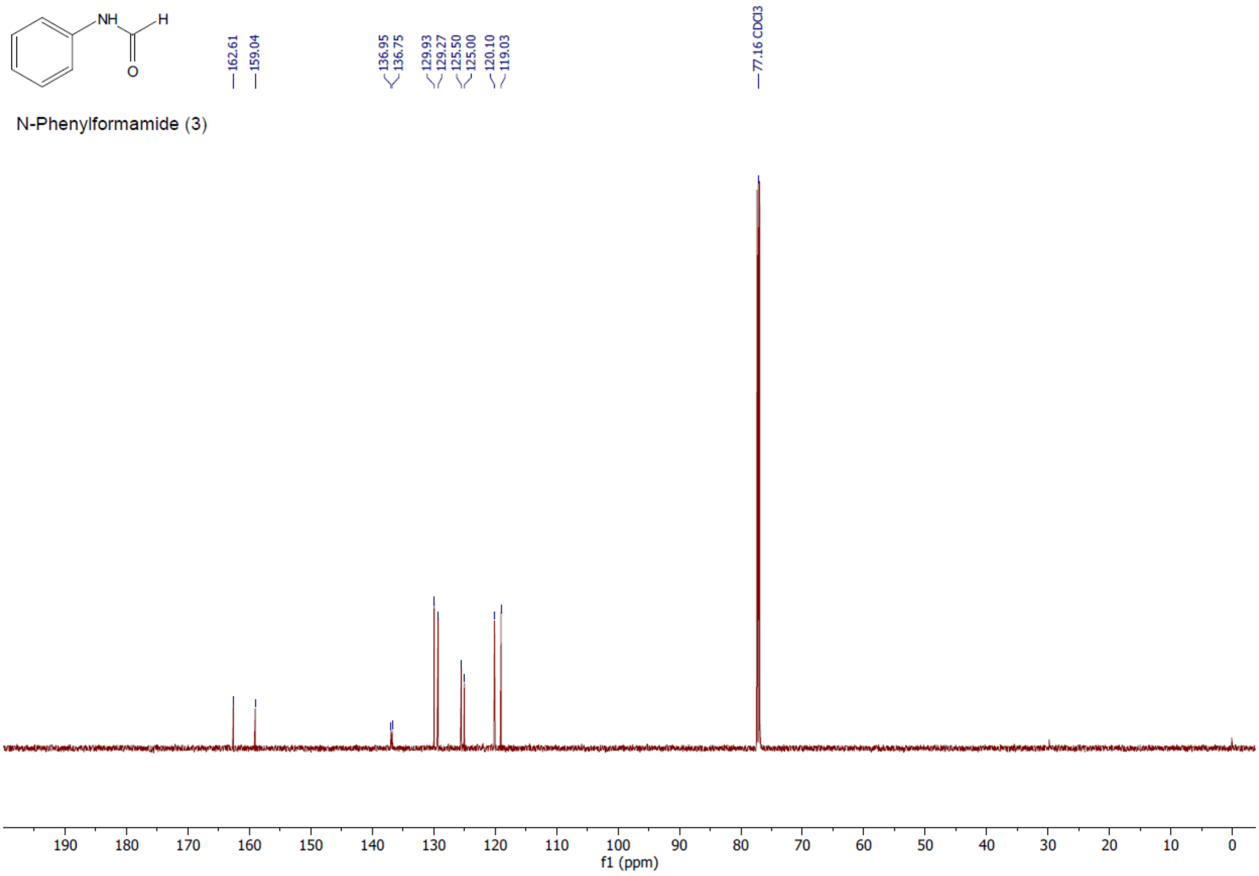

N-Phenylformamide (3)

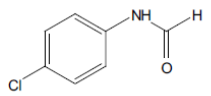

N-(4-Chlorophenyl)formamide (4)

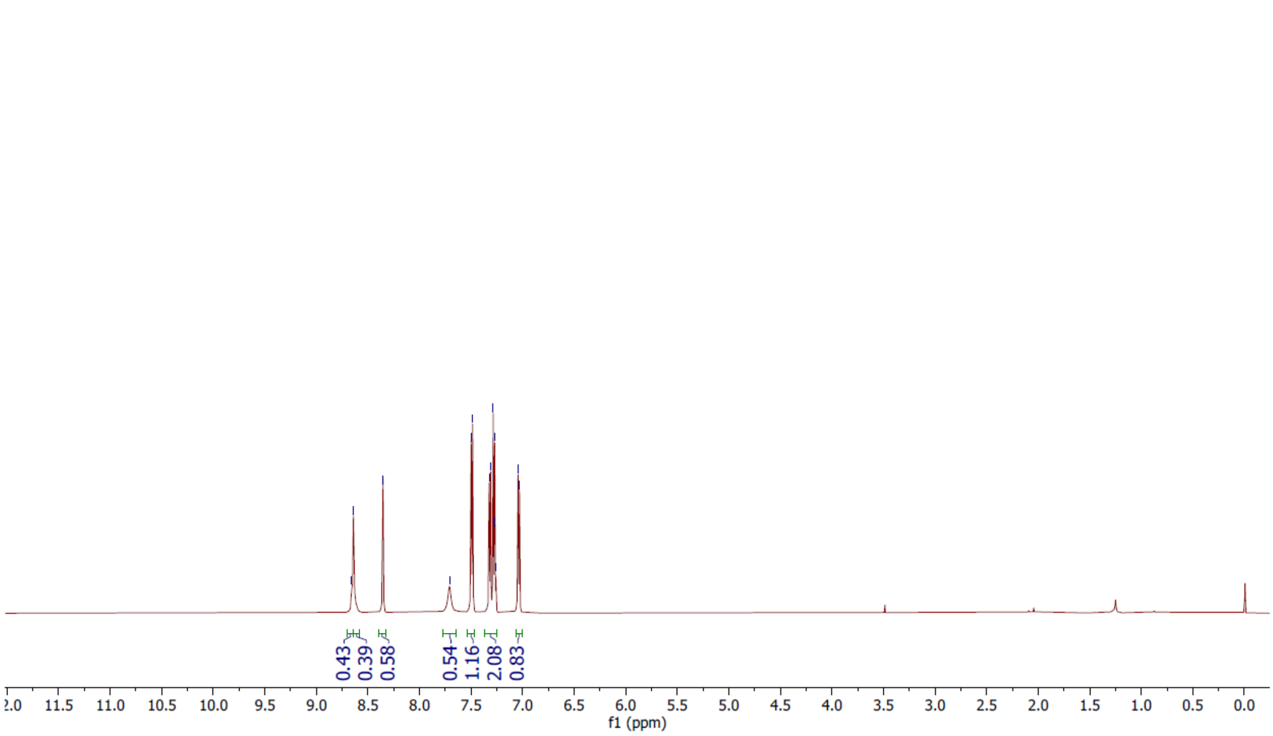

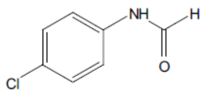

N-(4-Chlorophenyl)formamide (4)

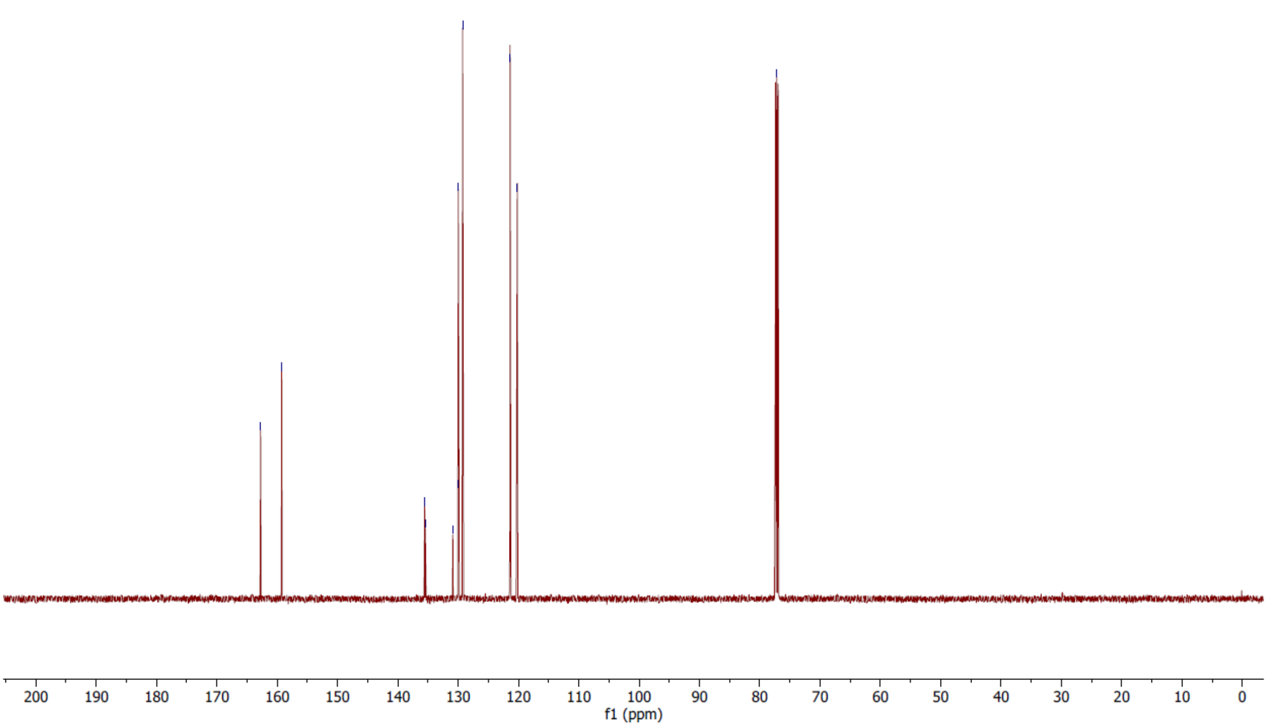

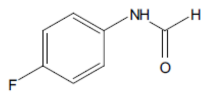

N-(4-Fluorophenyl)formamide (5)

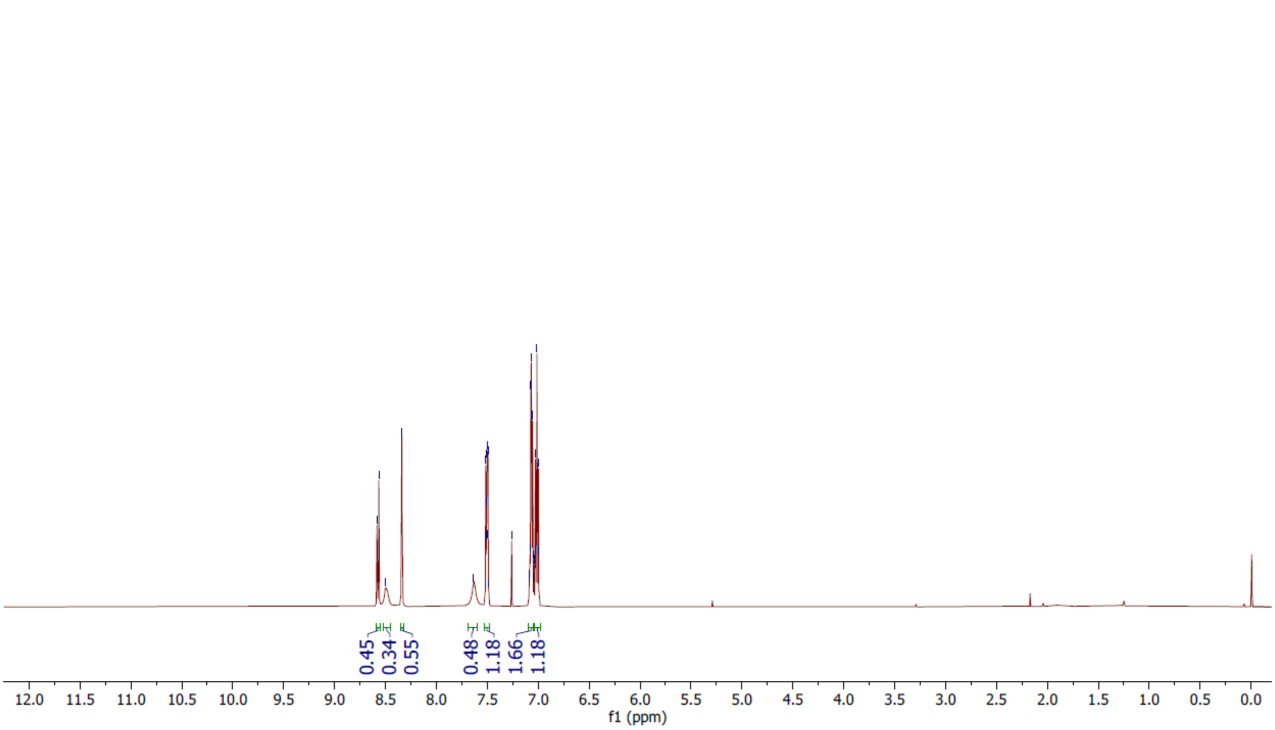

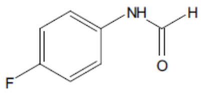

N-(4-Fluorophenyl)formamide (5)

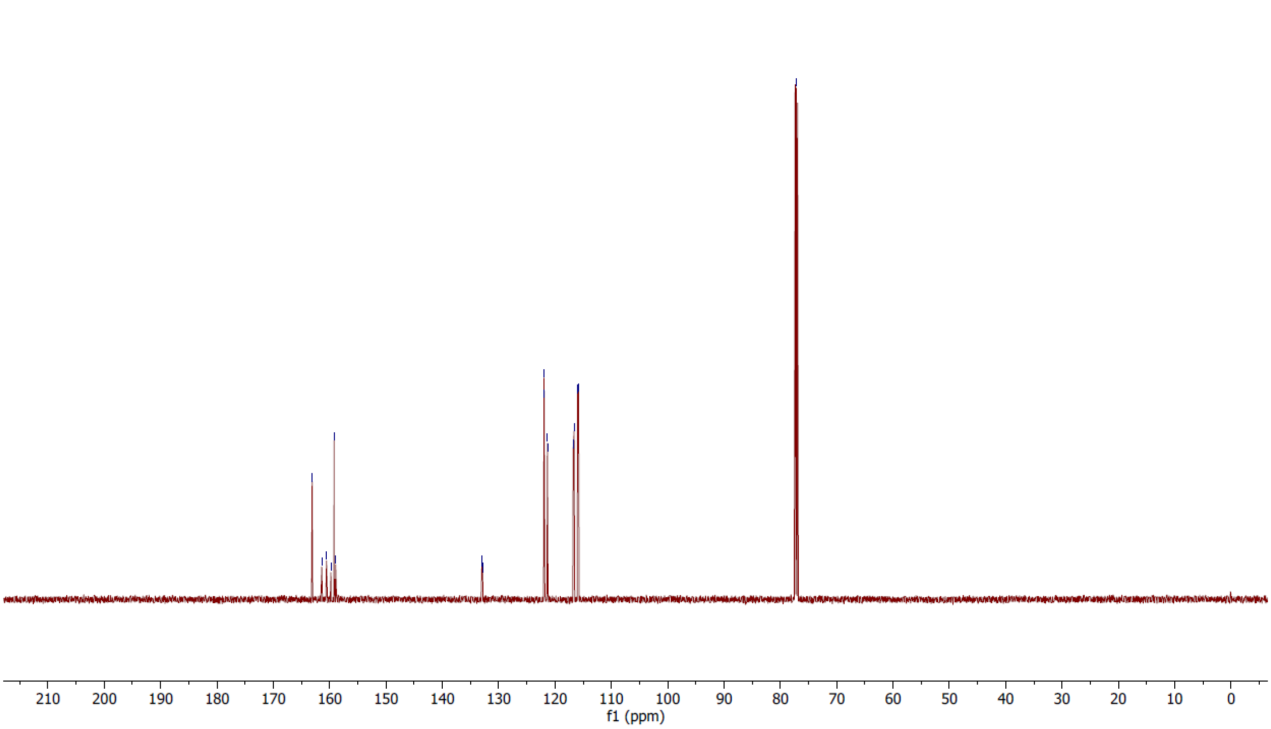

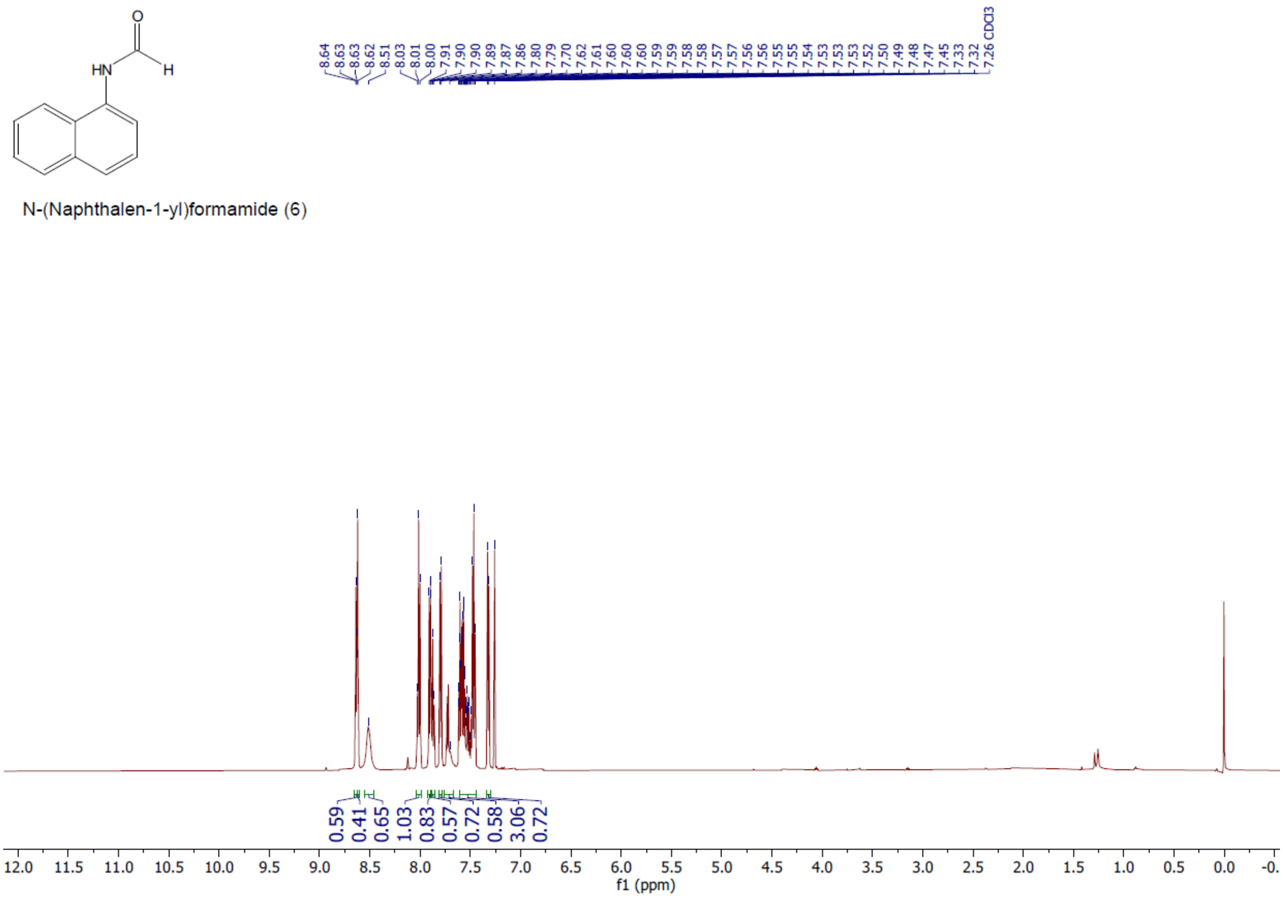

N-(Naphthalen-1-yl)formamide (6)

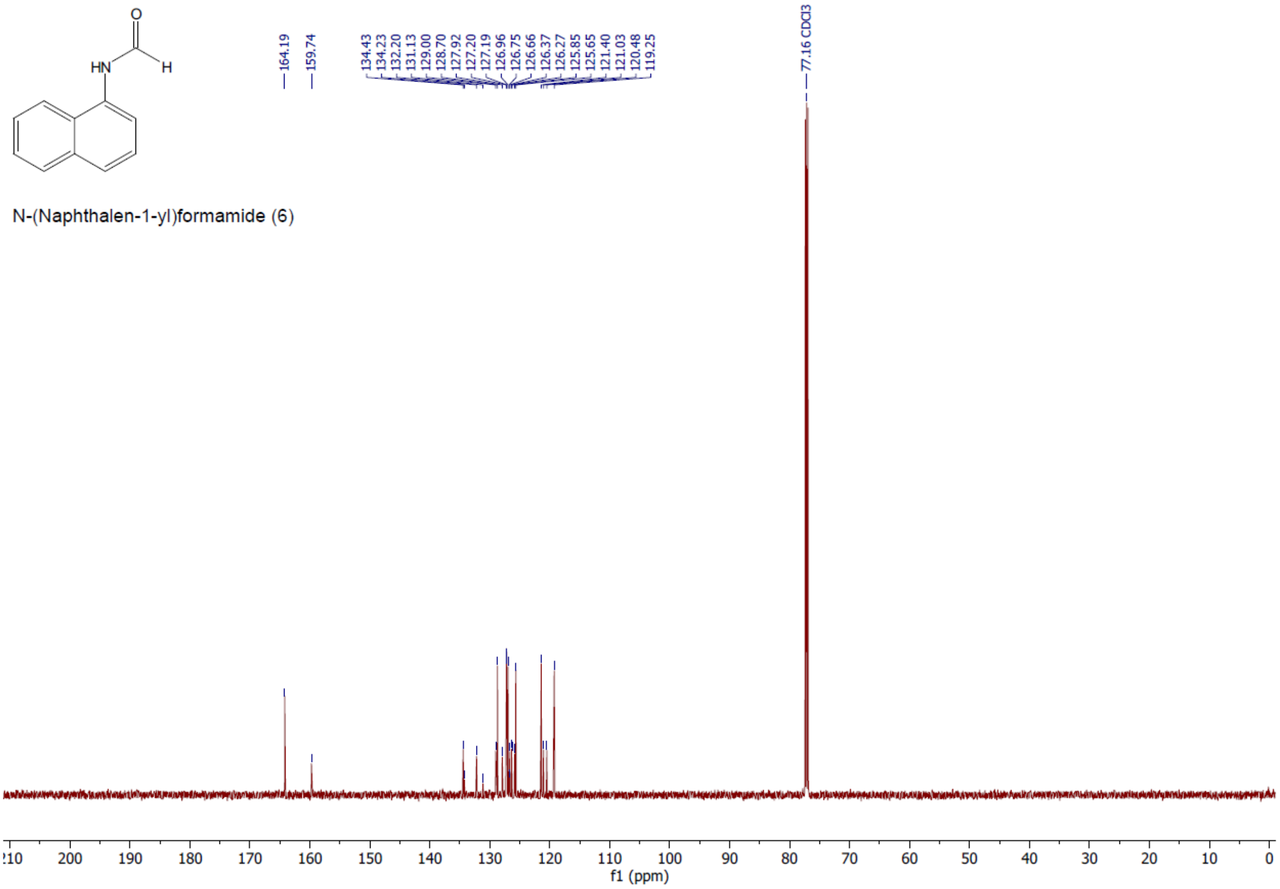

N-(Naphthalen-1-yl)formamide (6)

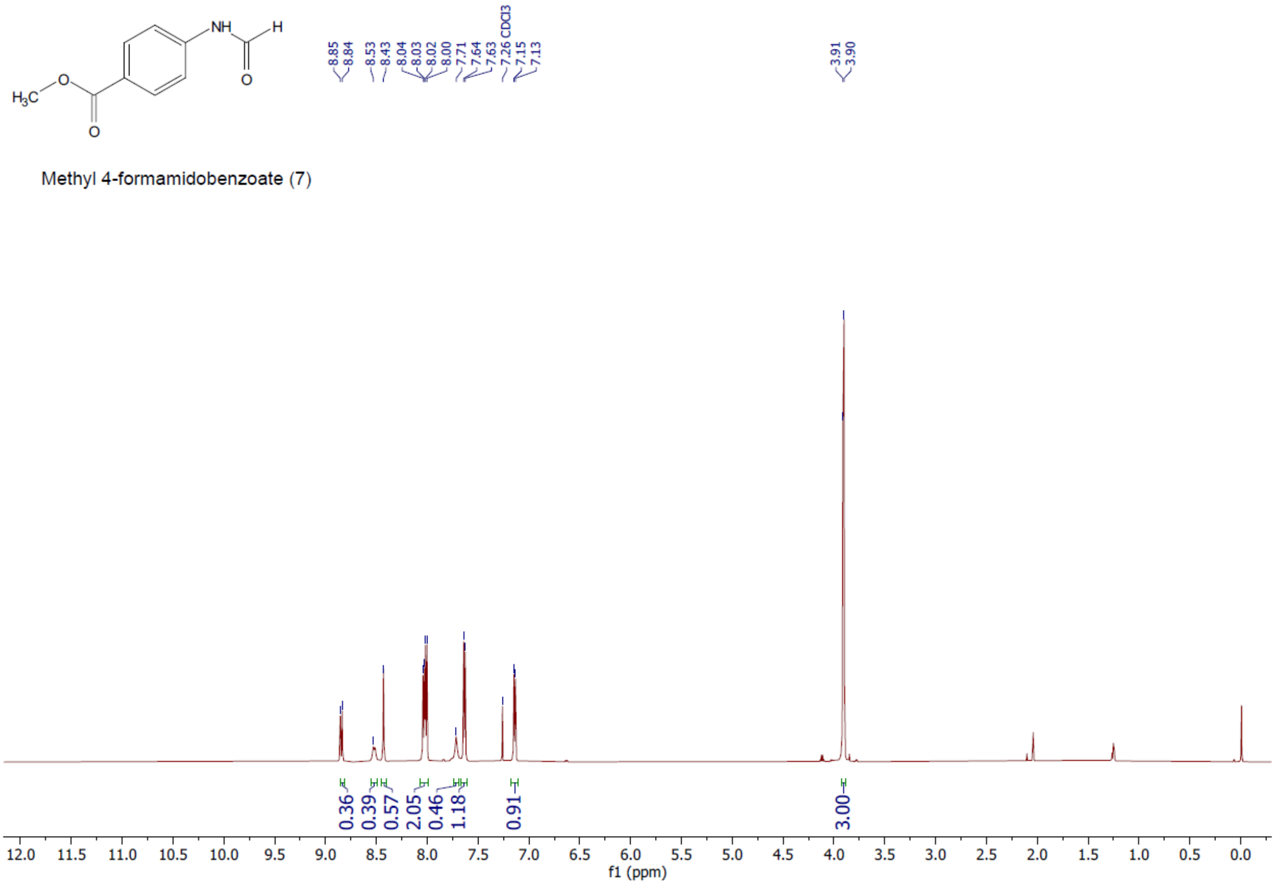

Methyl 4-formamidobenzoate (7)

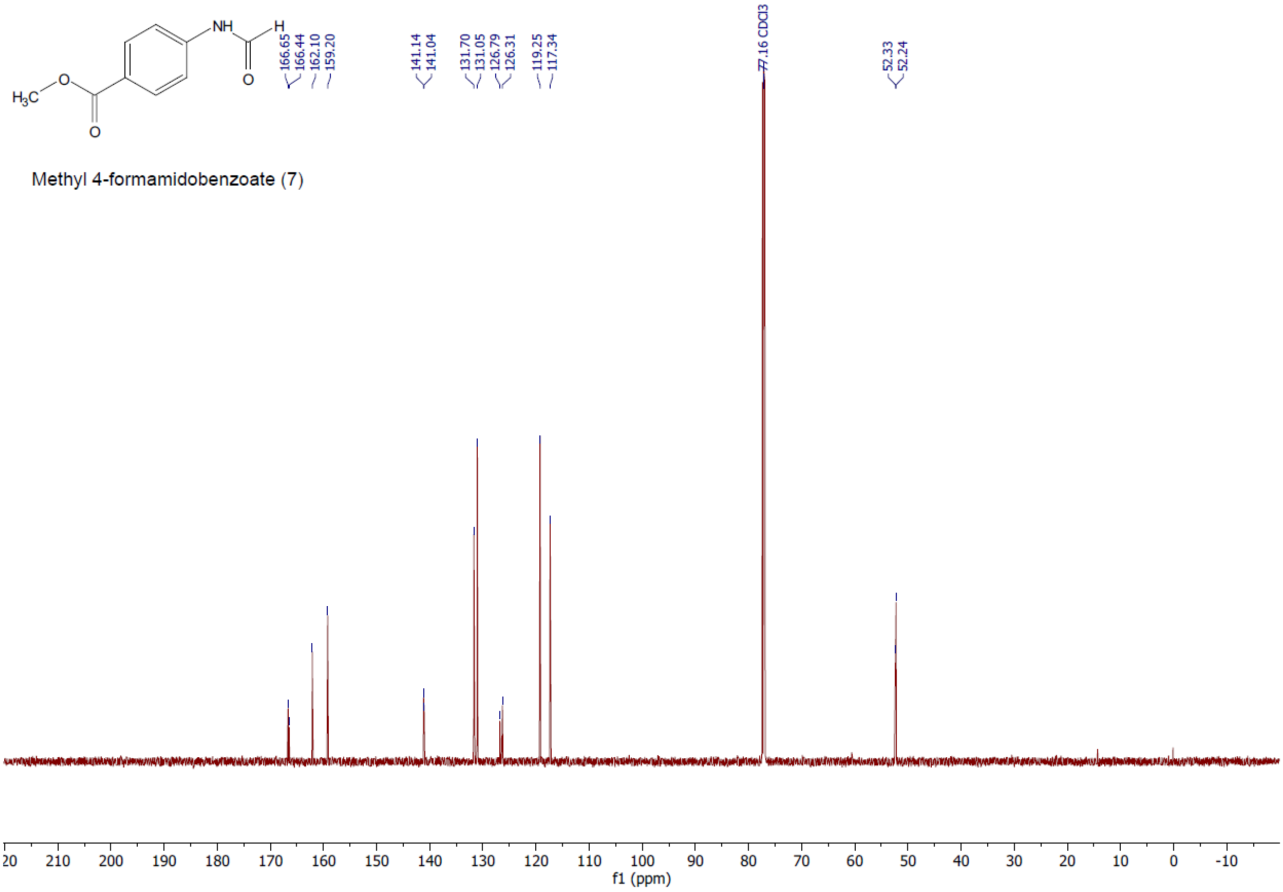

Methyl 4-formamidobenzoate (7)

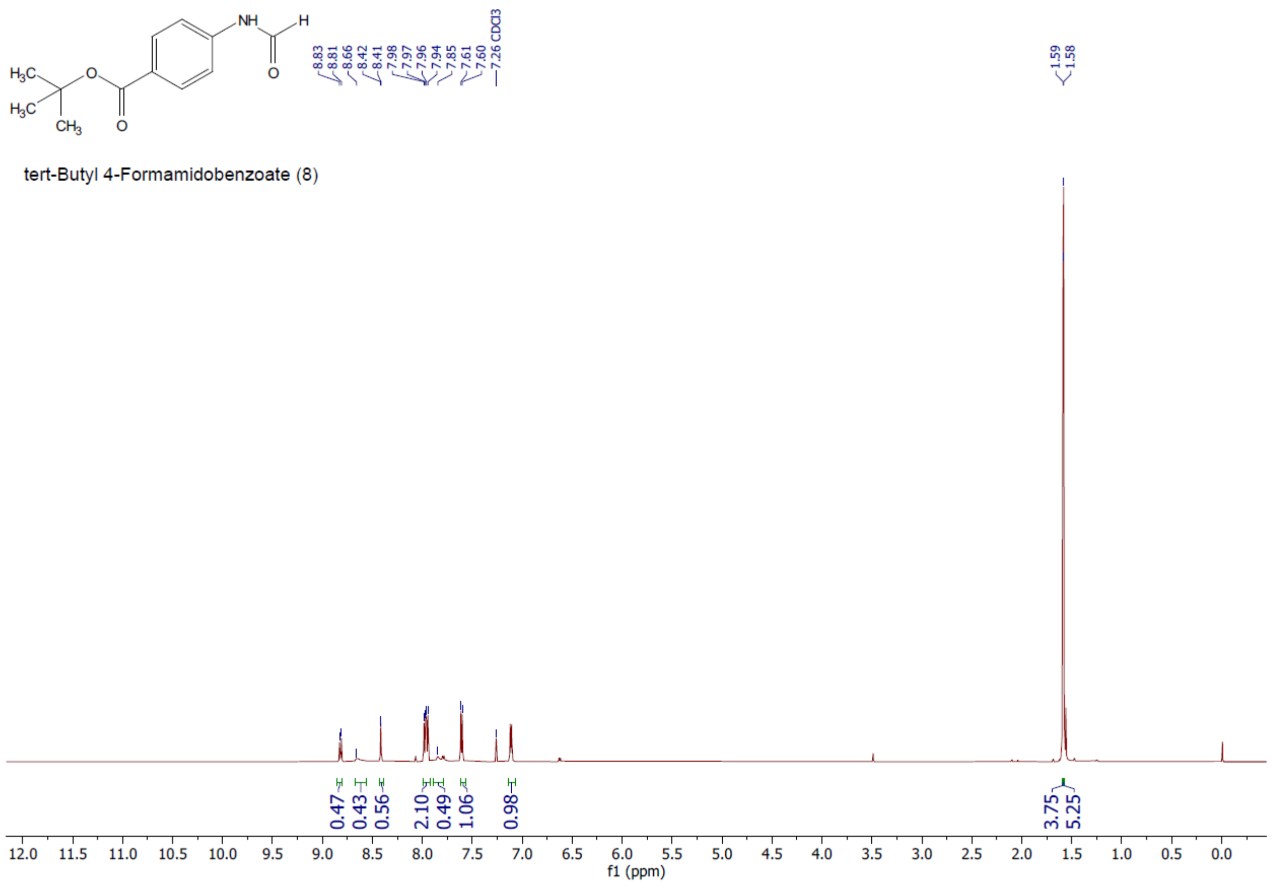

tert-Butyl 4-Formamidobenzoate (8)

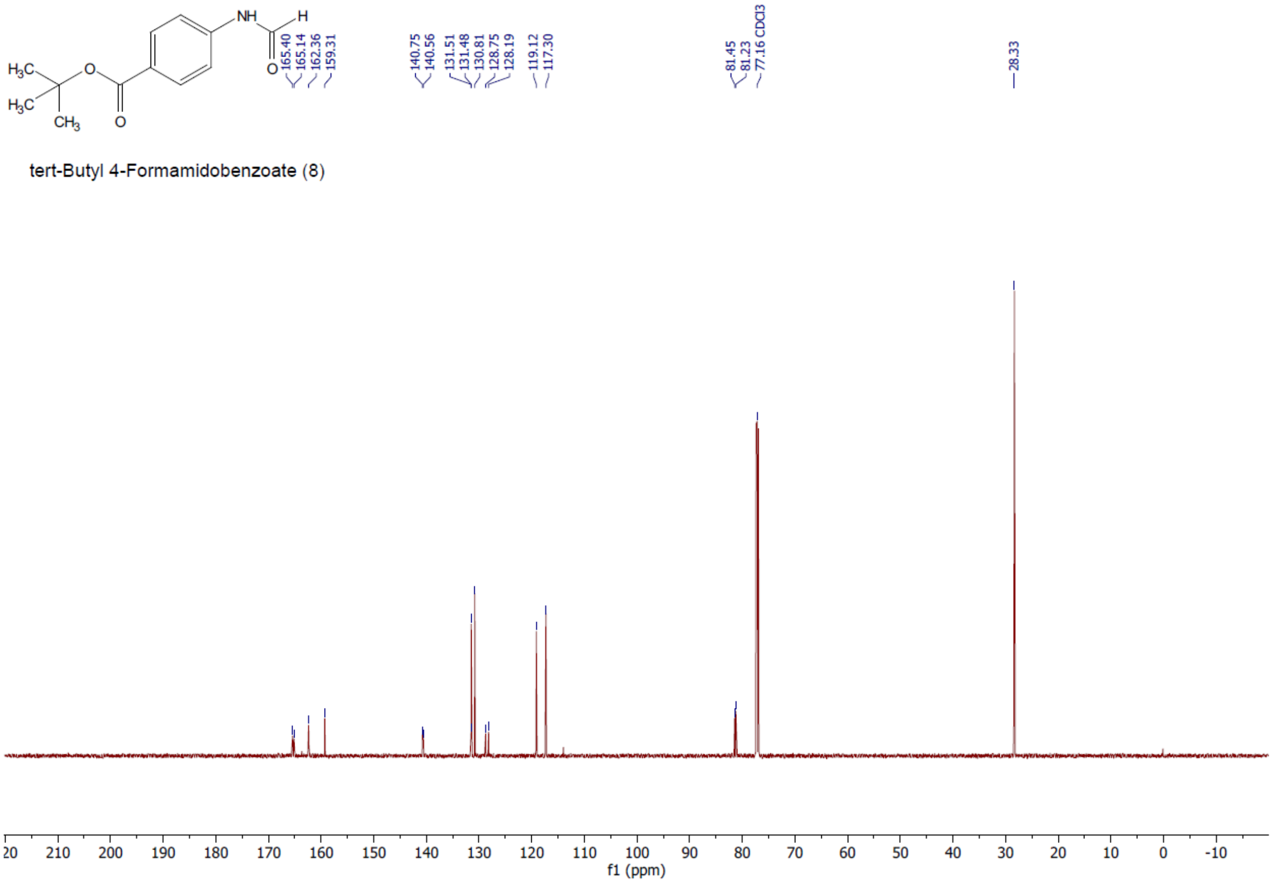

tert-Butyl 4-Formamidobenzoate (8)

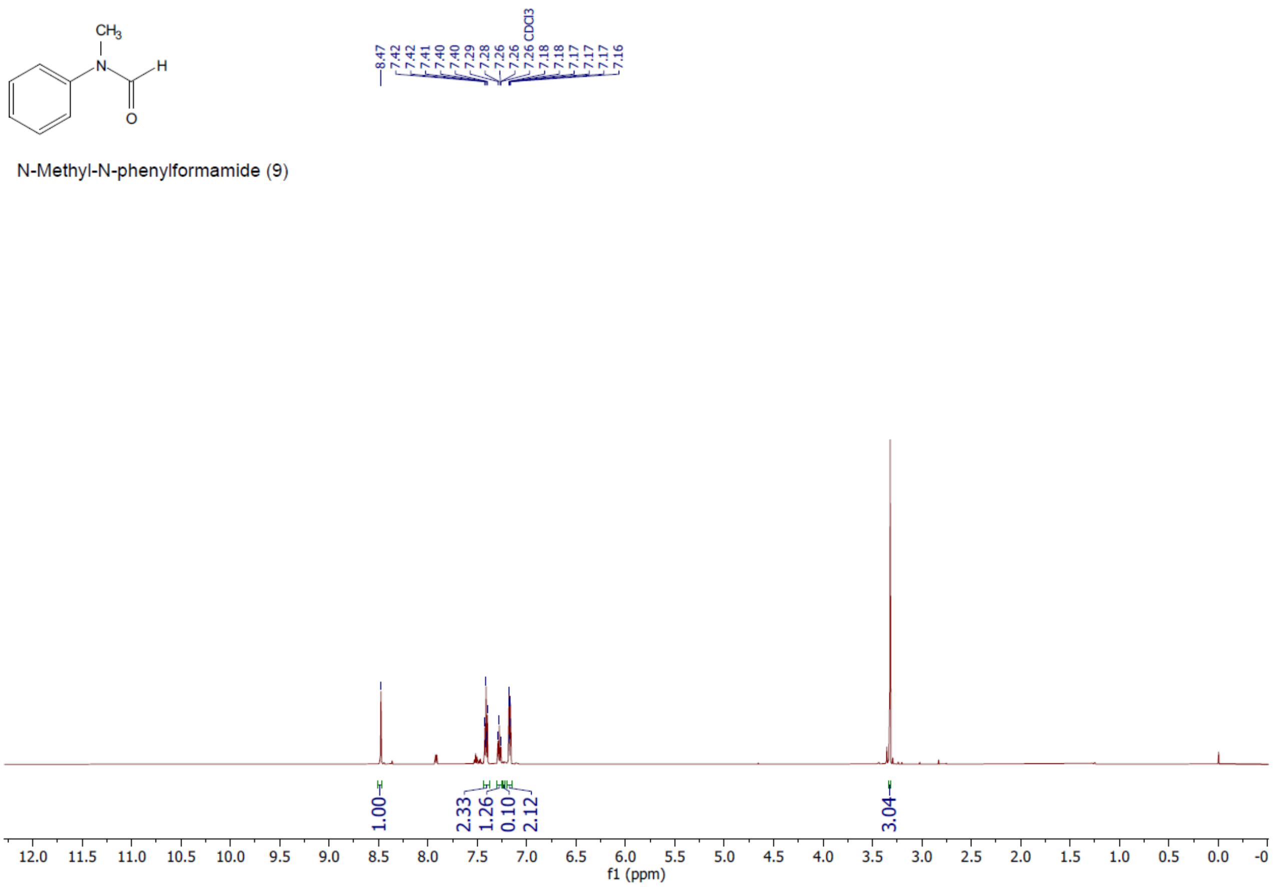

N-Methyl-N-phenylformamide (9)

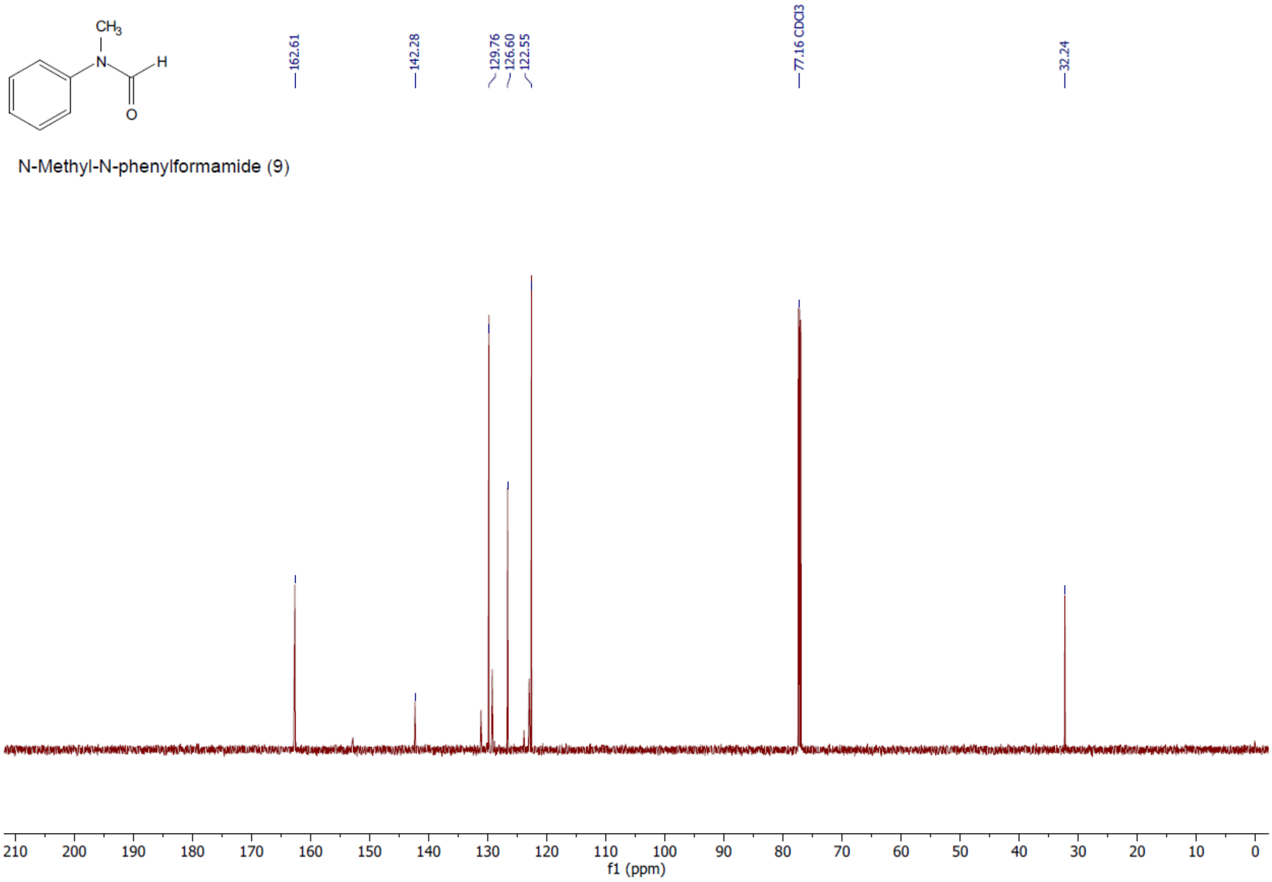

N-Methyl-N-phenylformamide (9)

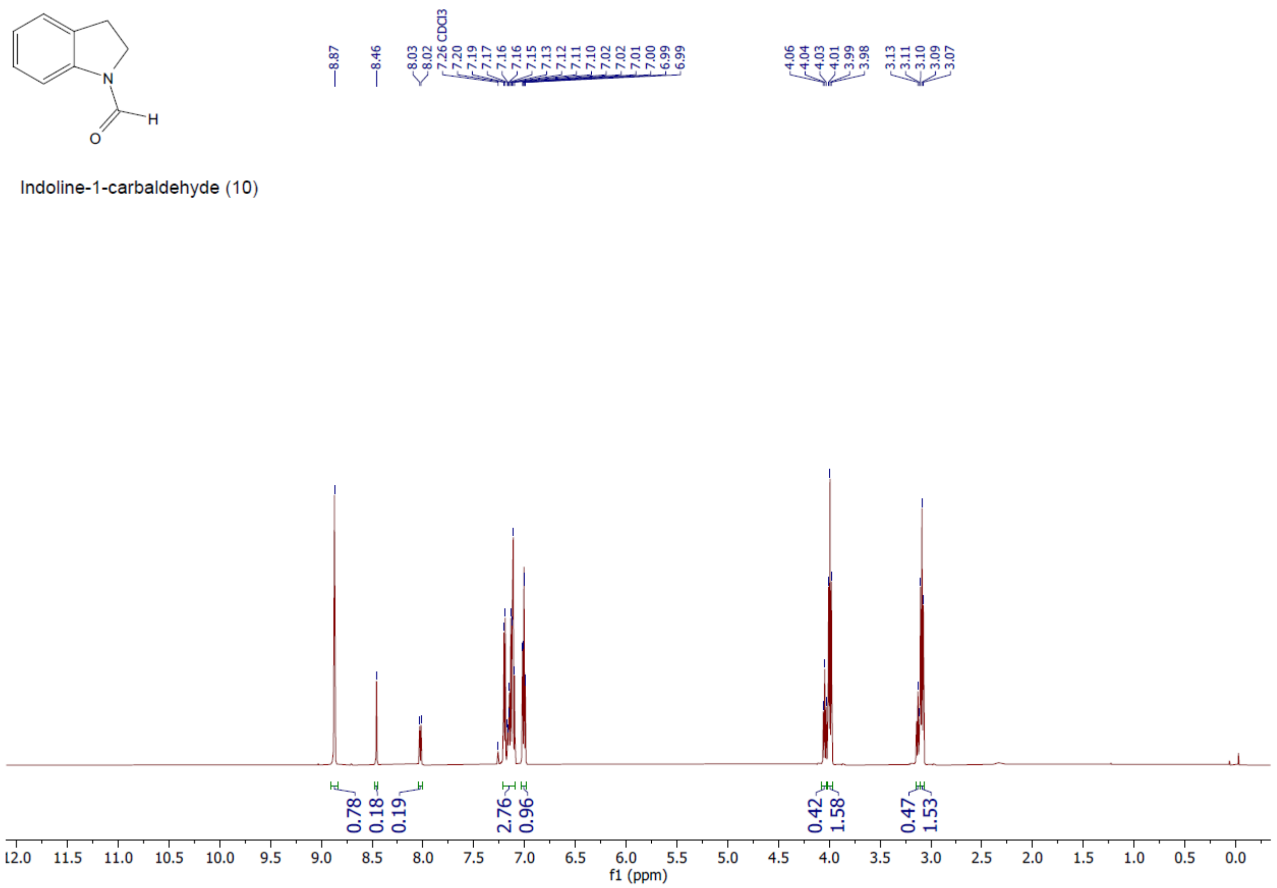

Indoline-1-carbaldehyde (10)

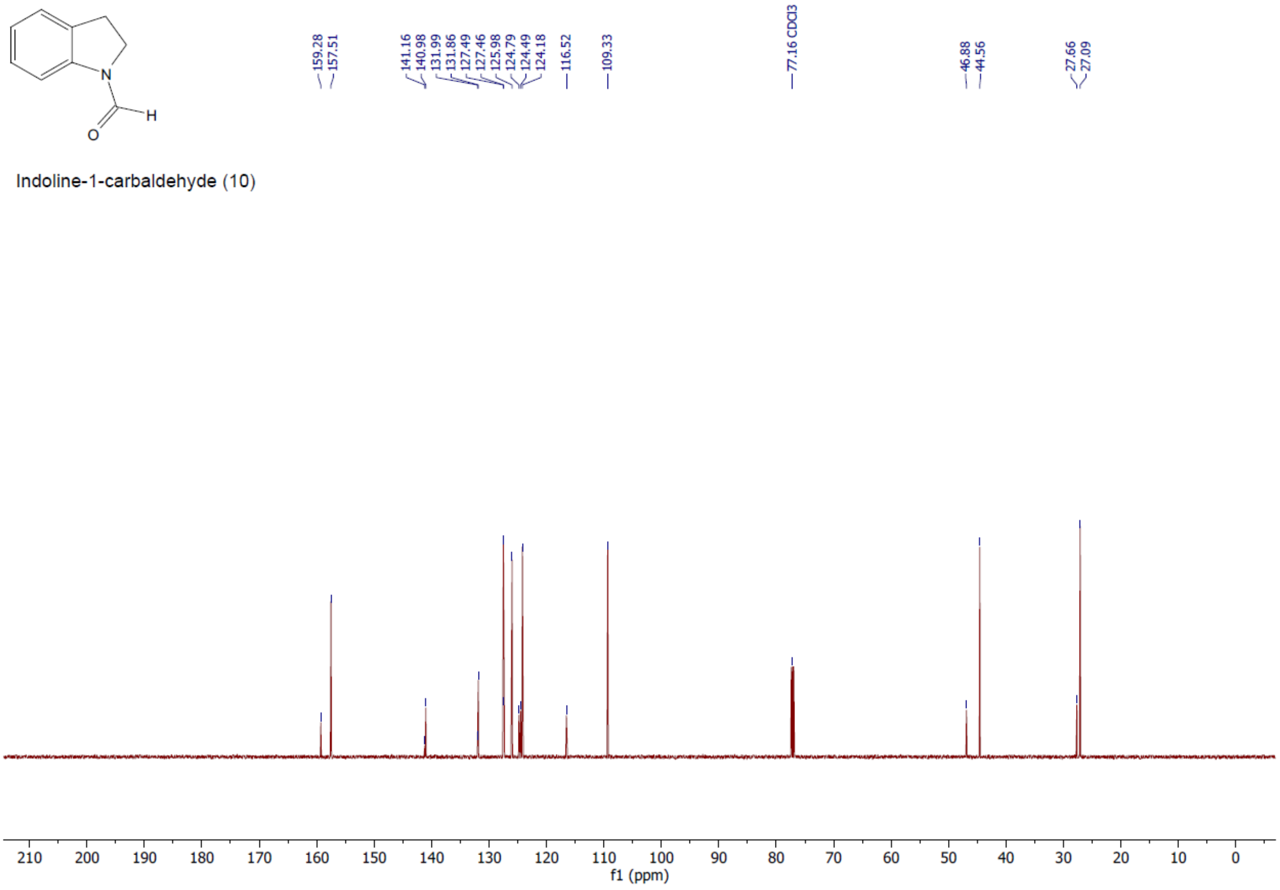

Indoline-1-carbaldehyde (10)

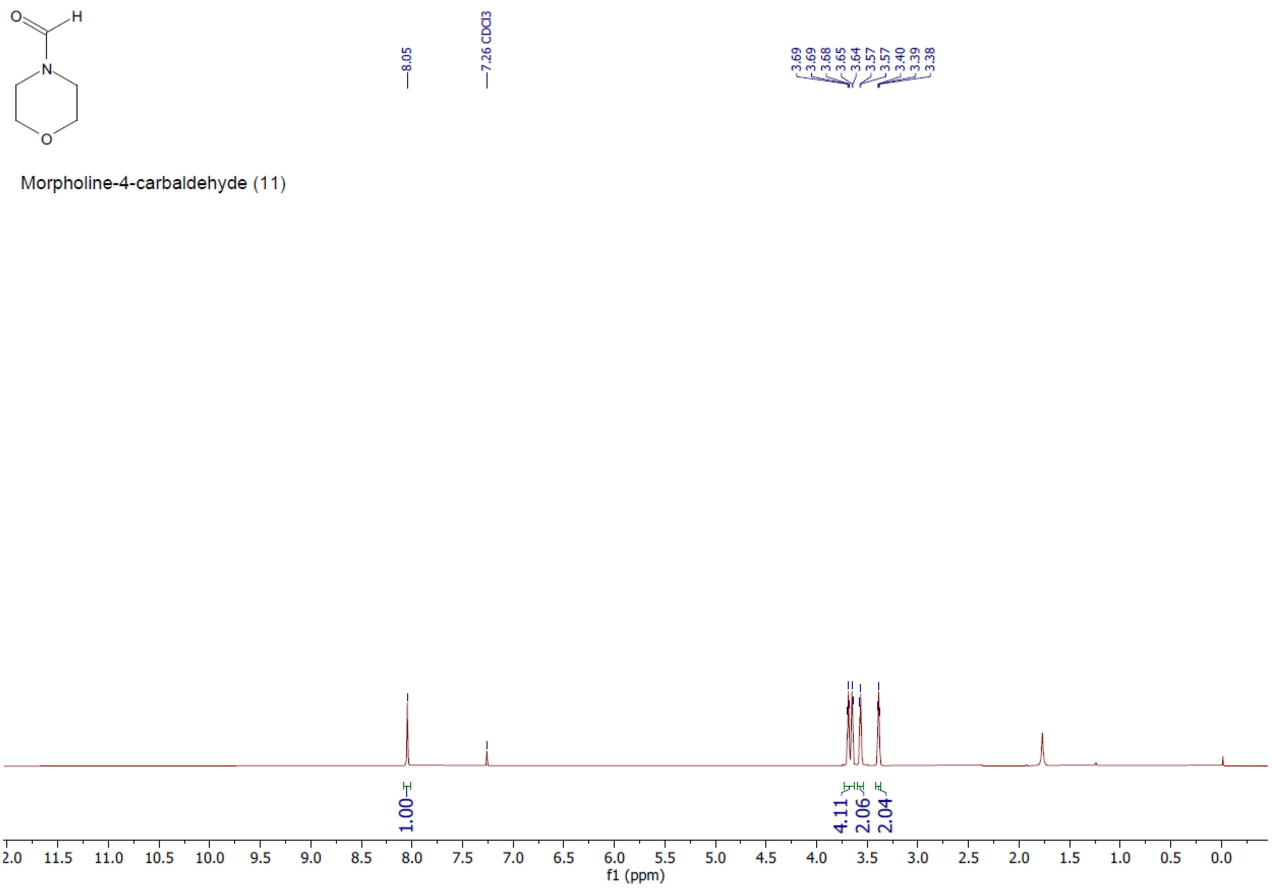

Morpholine-4-carbaldehyde (11)

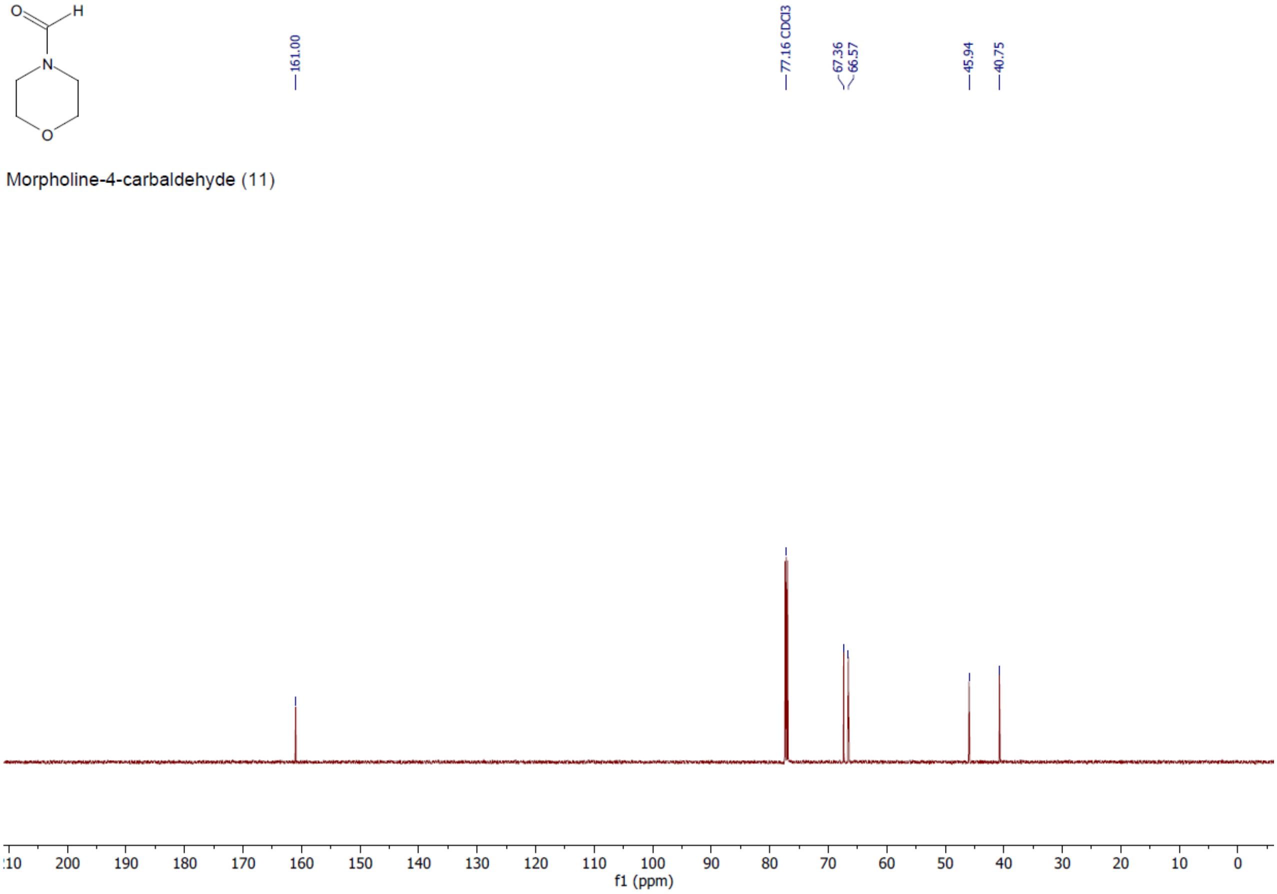

Morpholine-4-carbaldehyde (11)

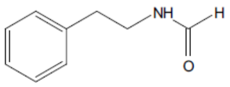

N-Phenethylformamide (13)

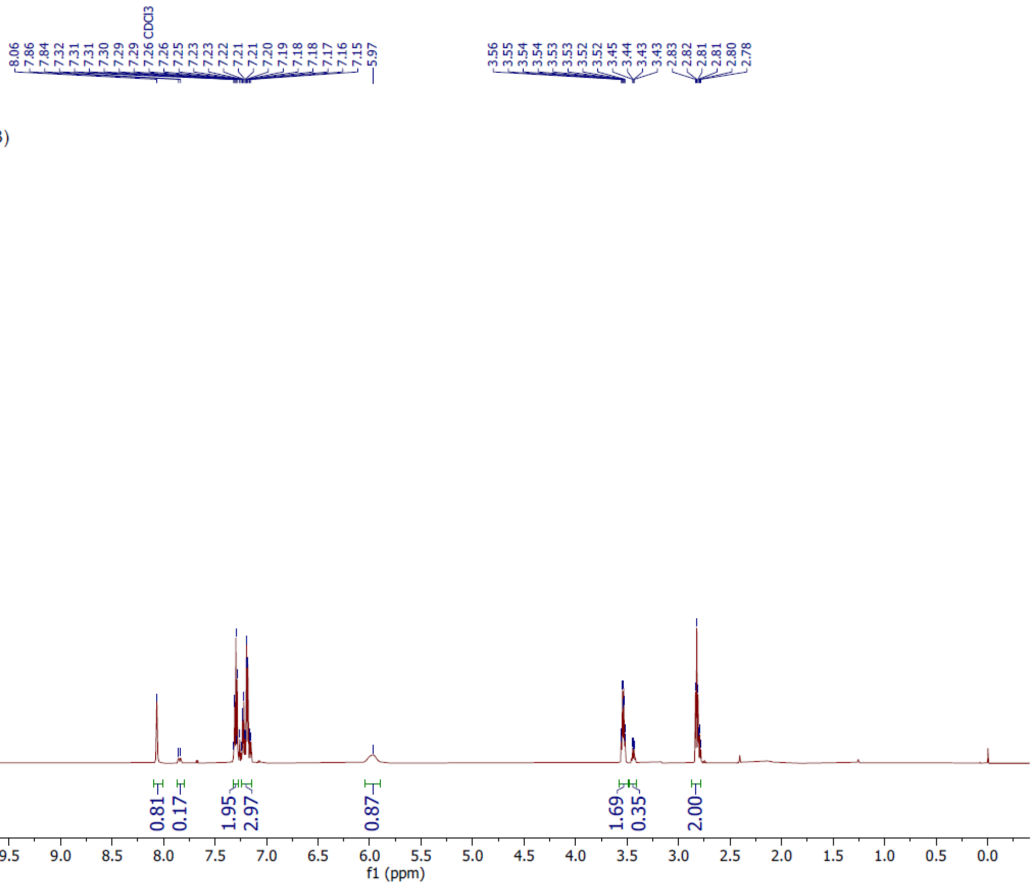

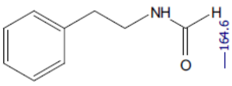

N-Phenethylformamide (13)

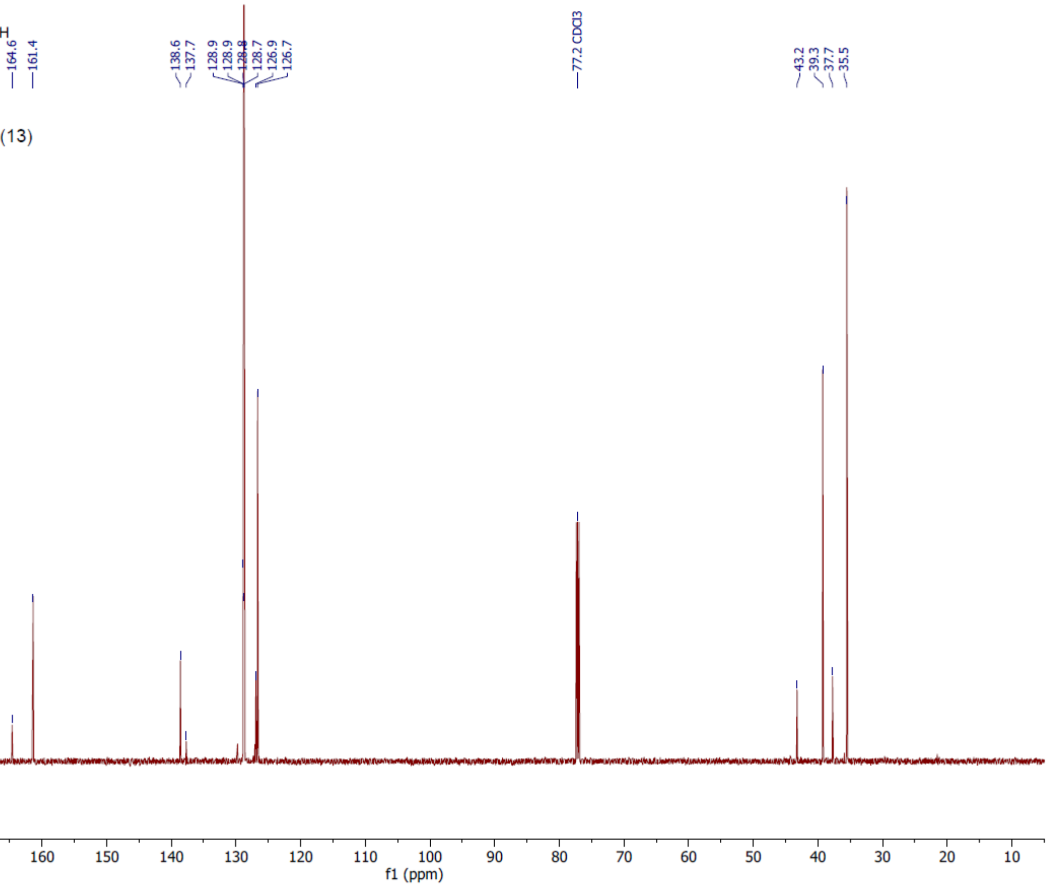

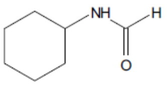

N-Cyclohexylformamide (14)

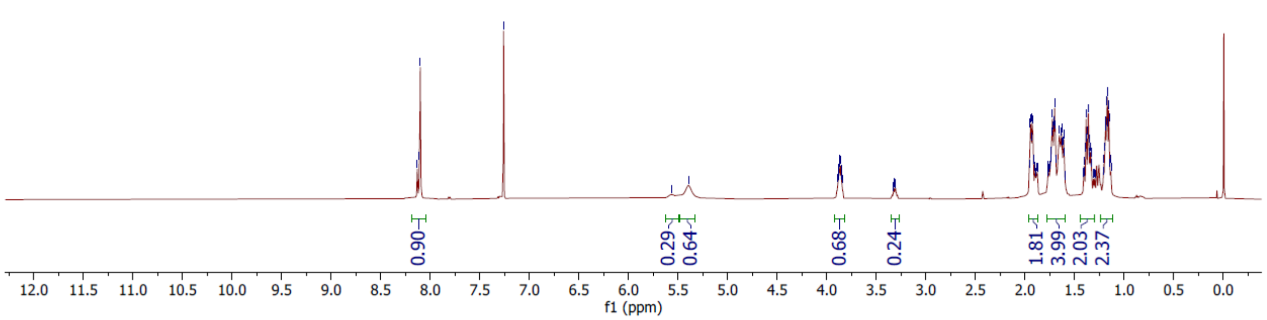

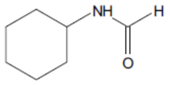

N-Cyclohexylformamide (14)

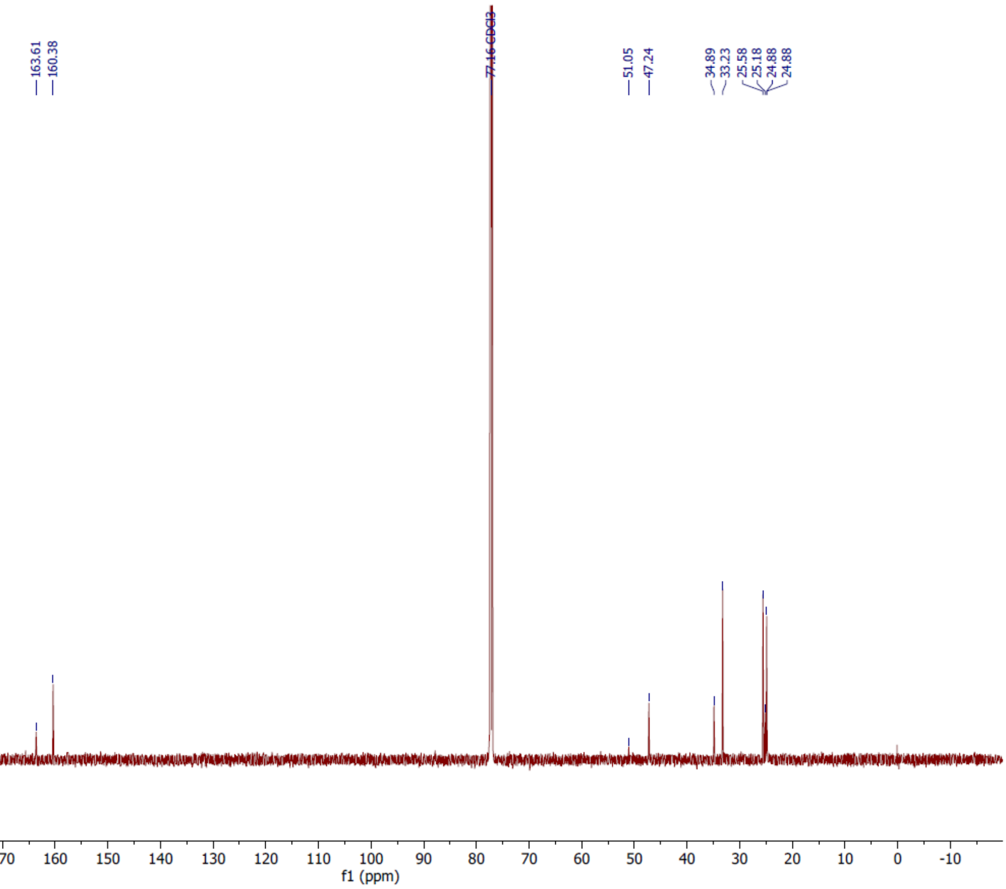

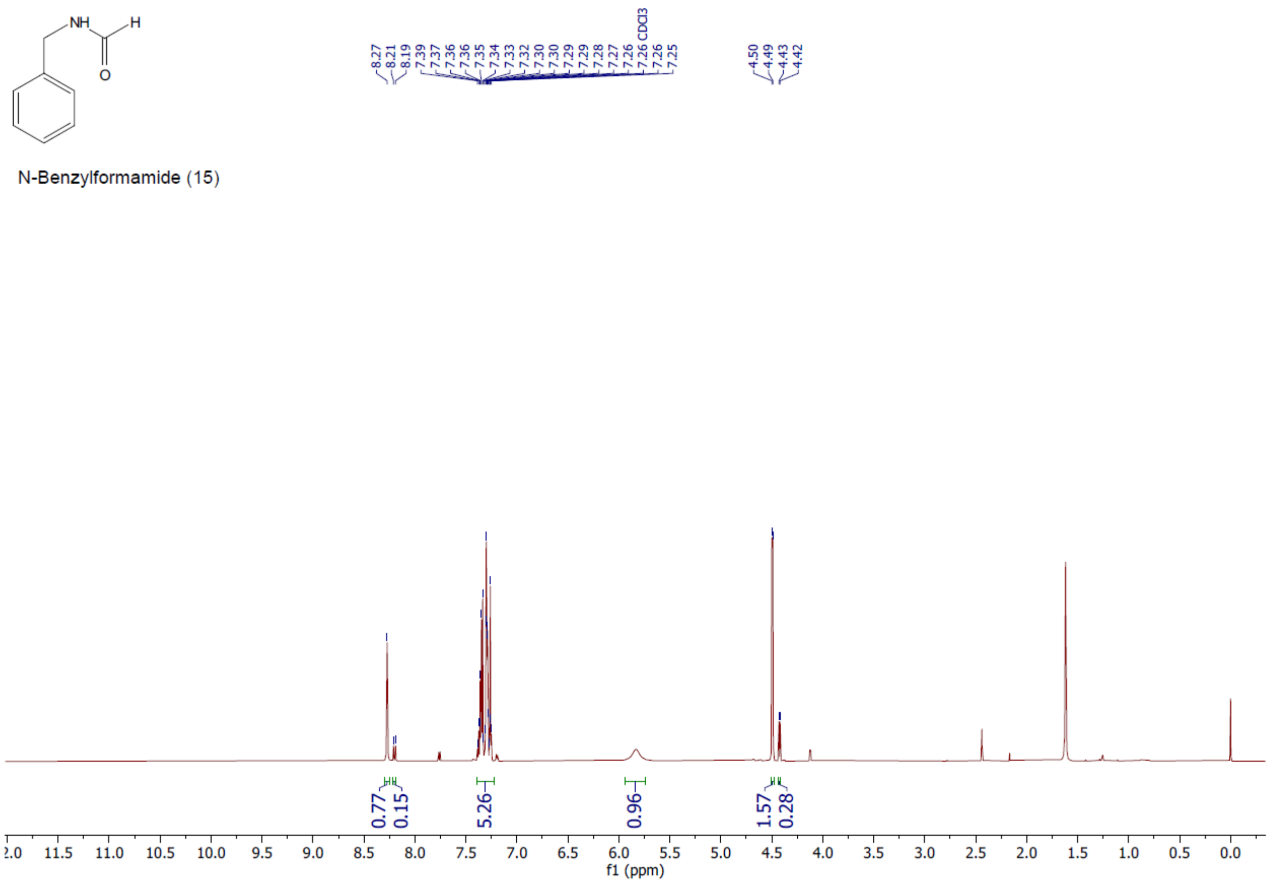

N-Benzylformamide (15)

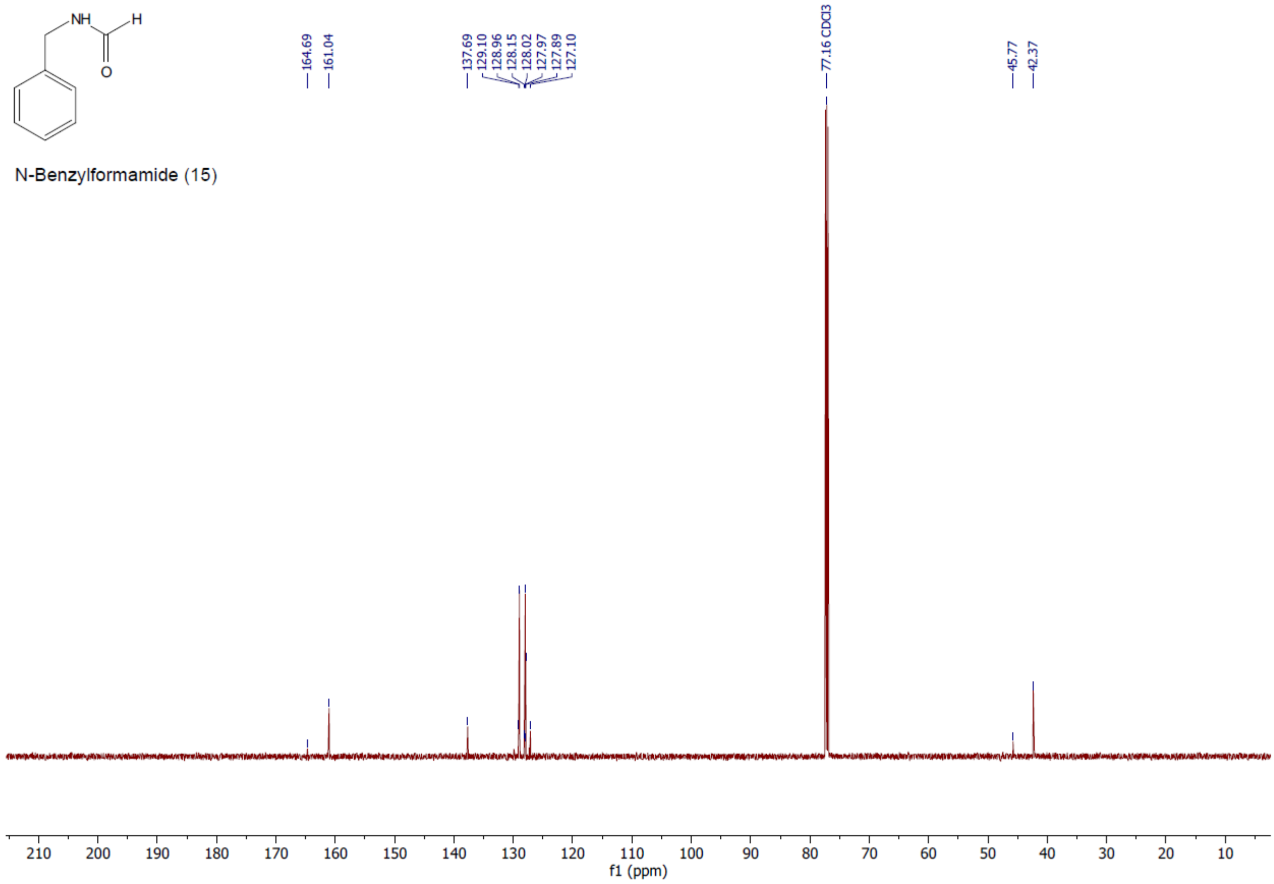

N-Benzylformamide (15)

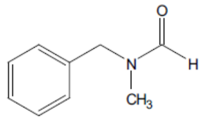

N-benzyl-N-methylformamide (16)

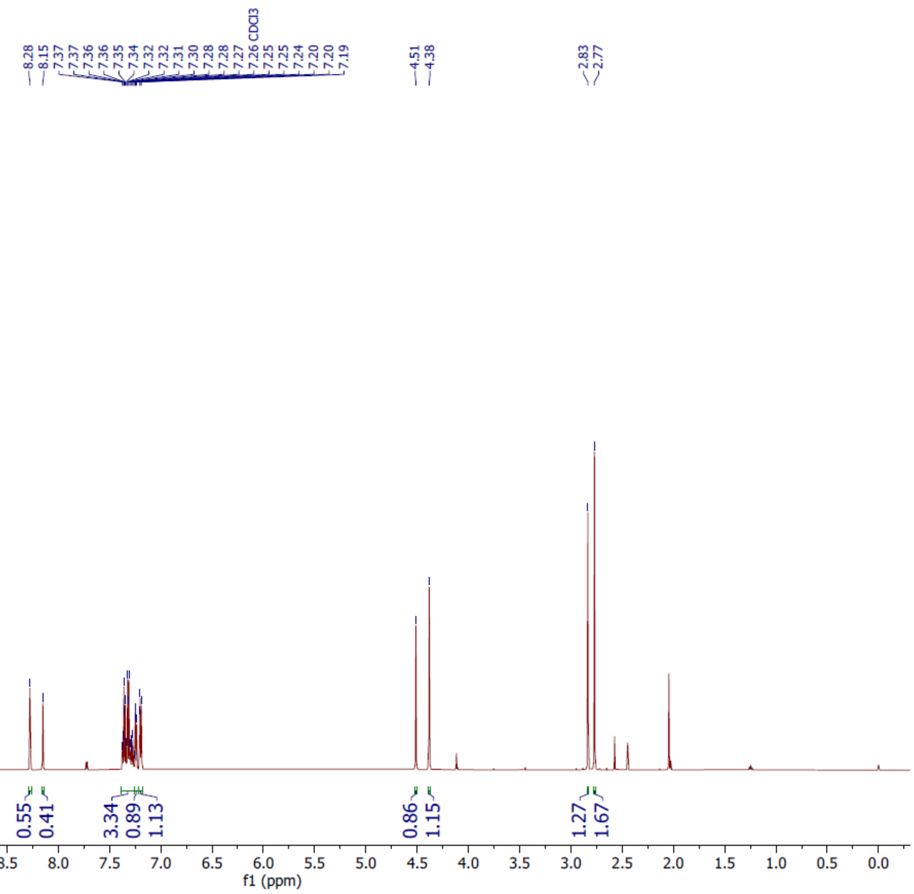

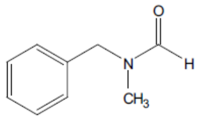

N-benzyl-N-methylformamide (16)

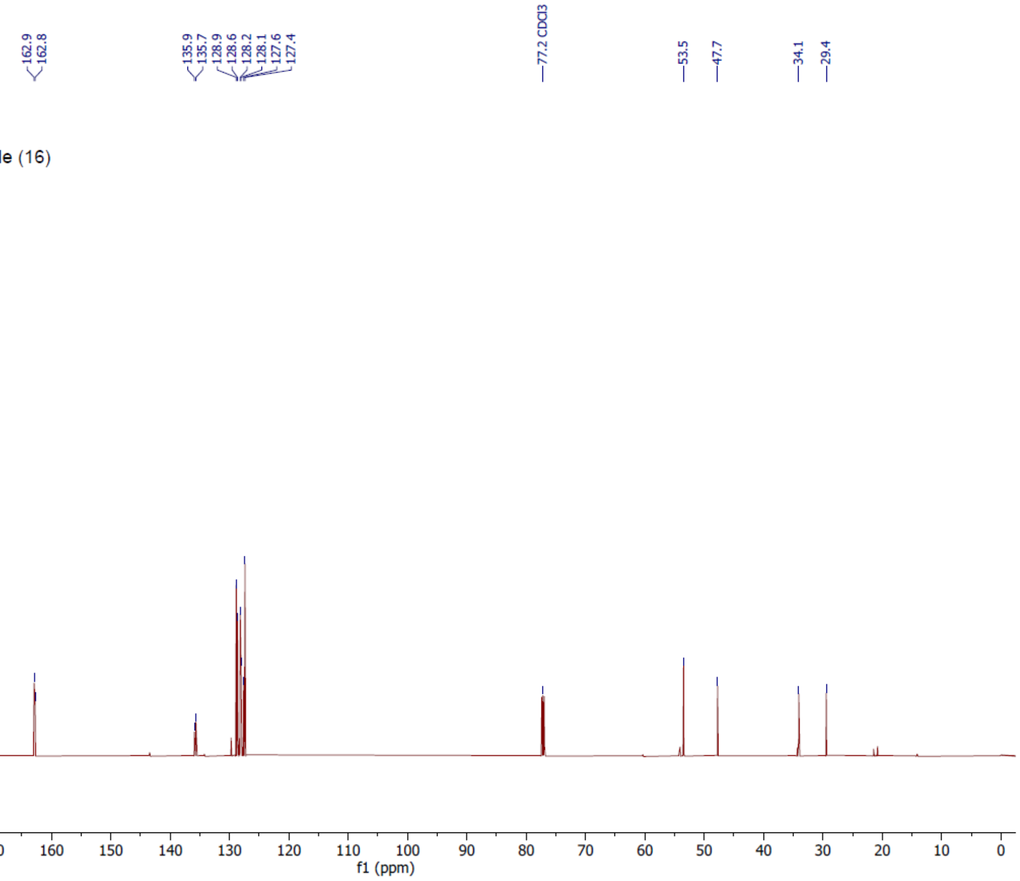

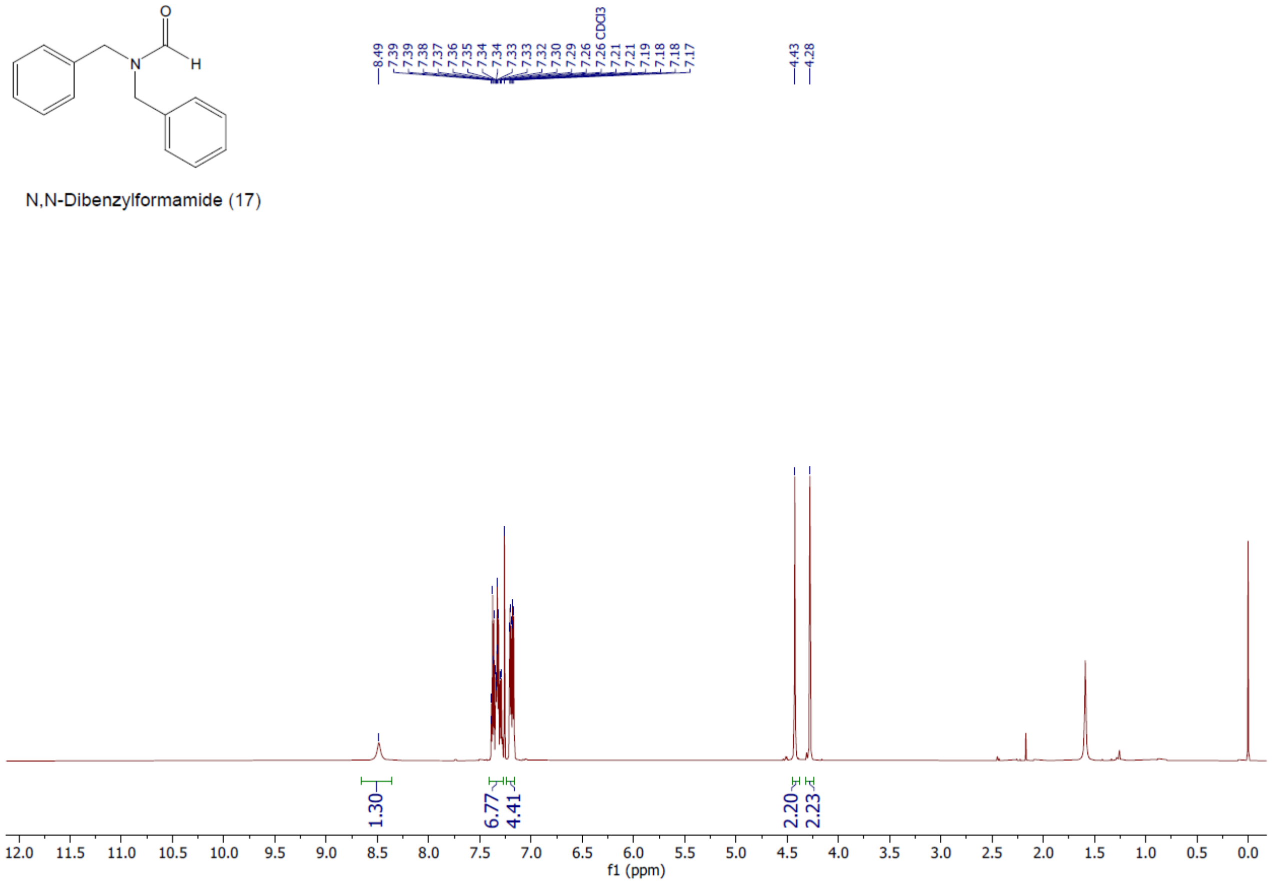

N,N-Dibenzylformamide (17)

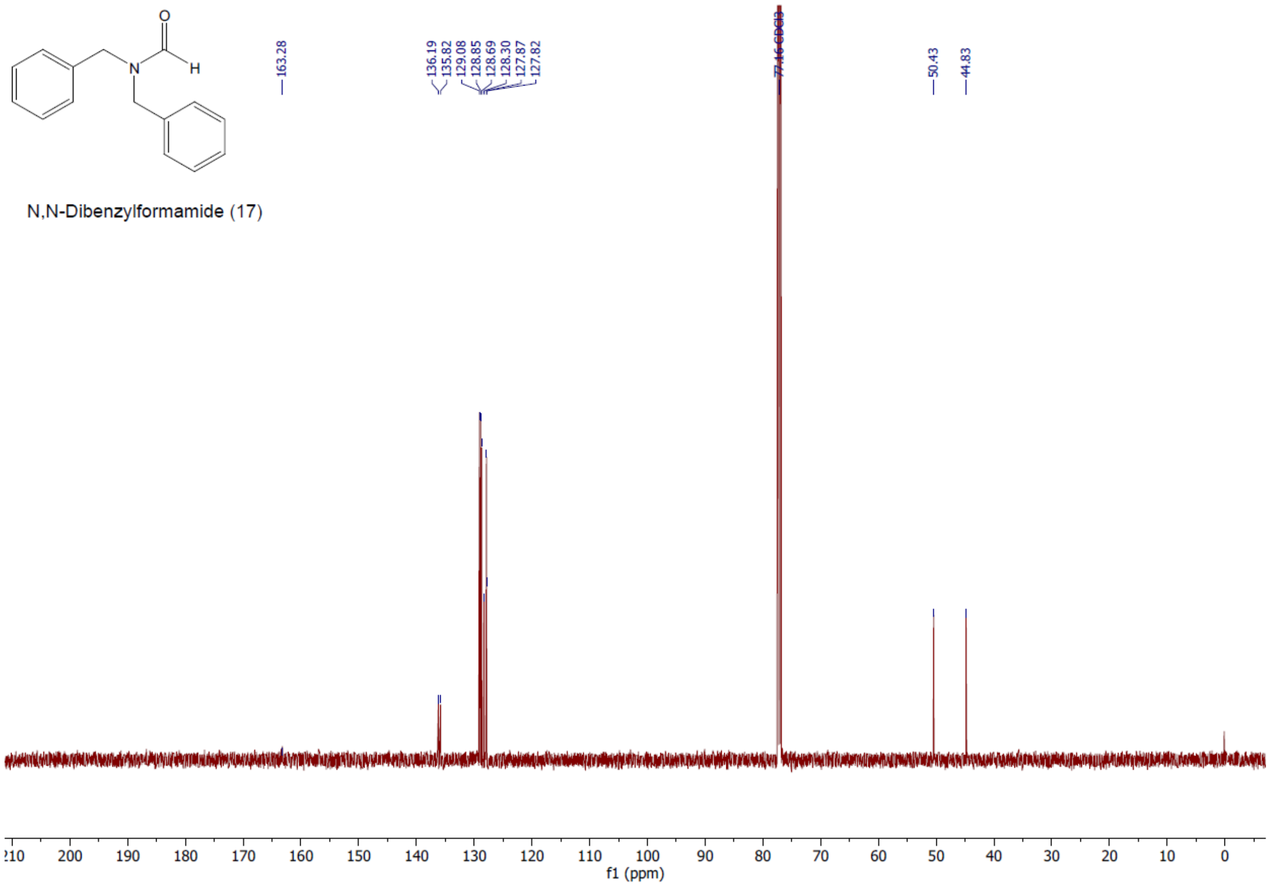

N,N-Dibenzylformamide (17)

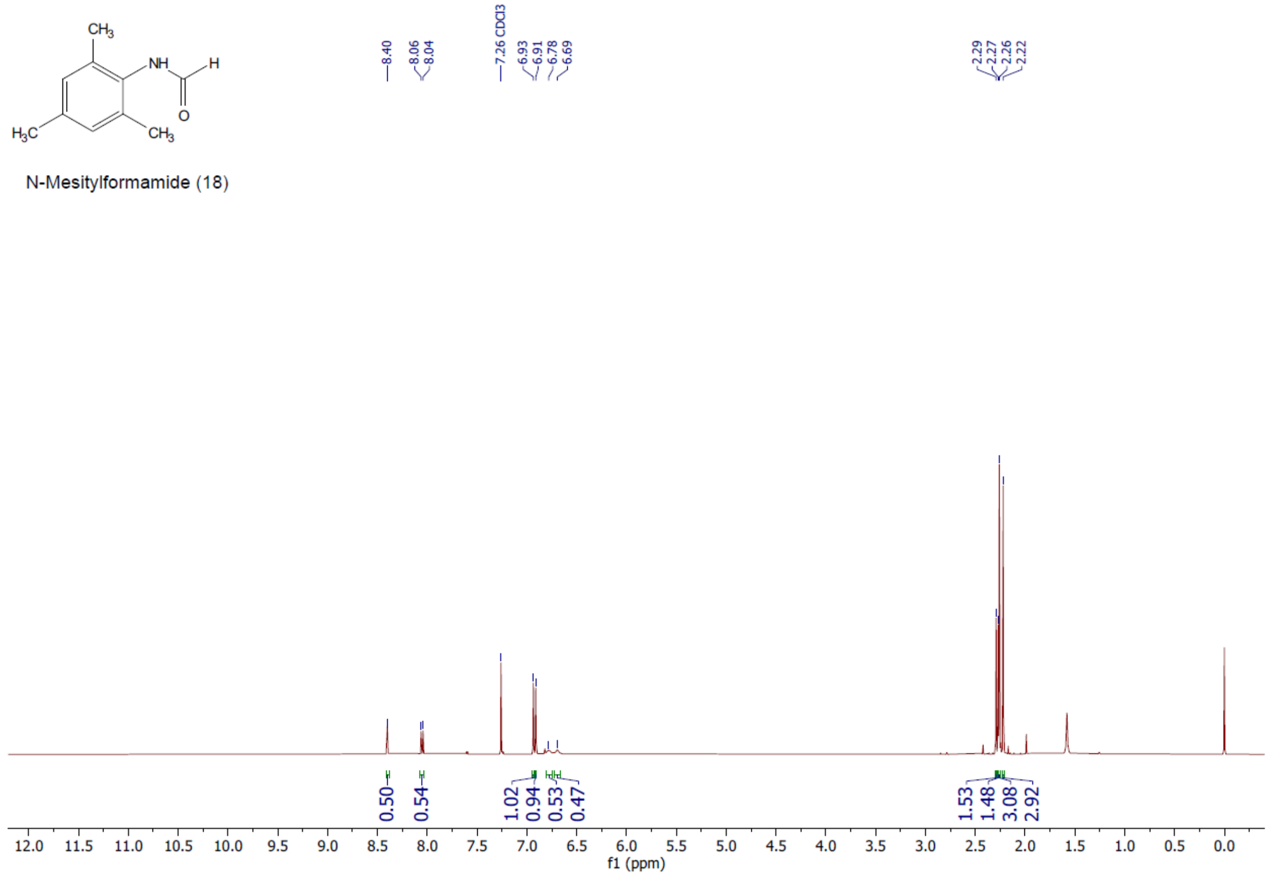

CH3
NH
H
O
H3C
CH3
N-Mesitylformamide (18)
8.40
8.06
8.04
7.26 CDCl3
6.93
6.91
6.78
6.69
2.29
2.27
2.26
2.22
0.50
0.54
1.02
0.94
0.53
0.47
1.53
1.48
3.08
2.92
f1 (ppm)

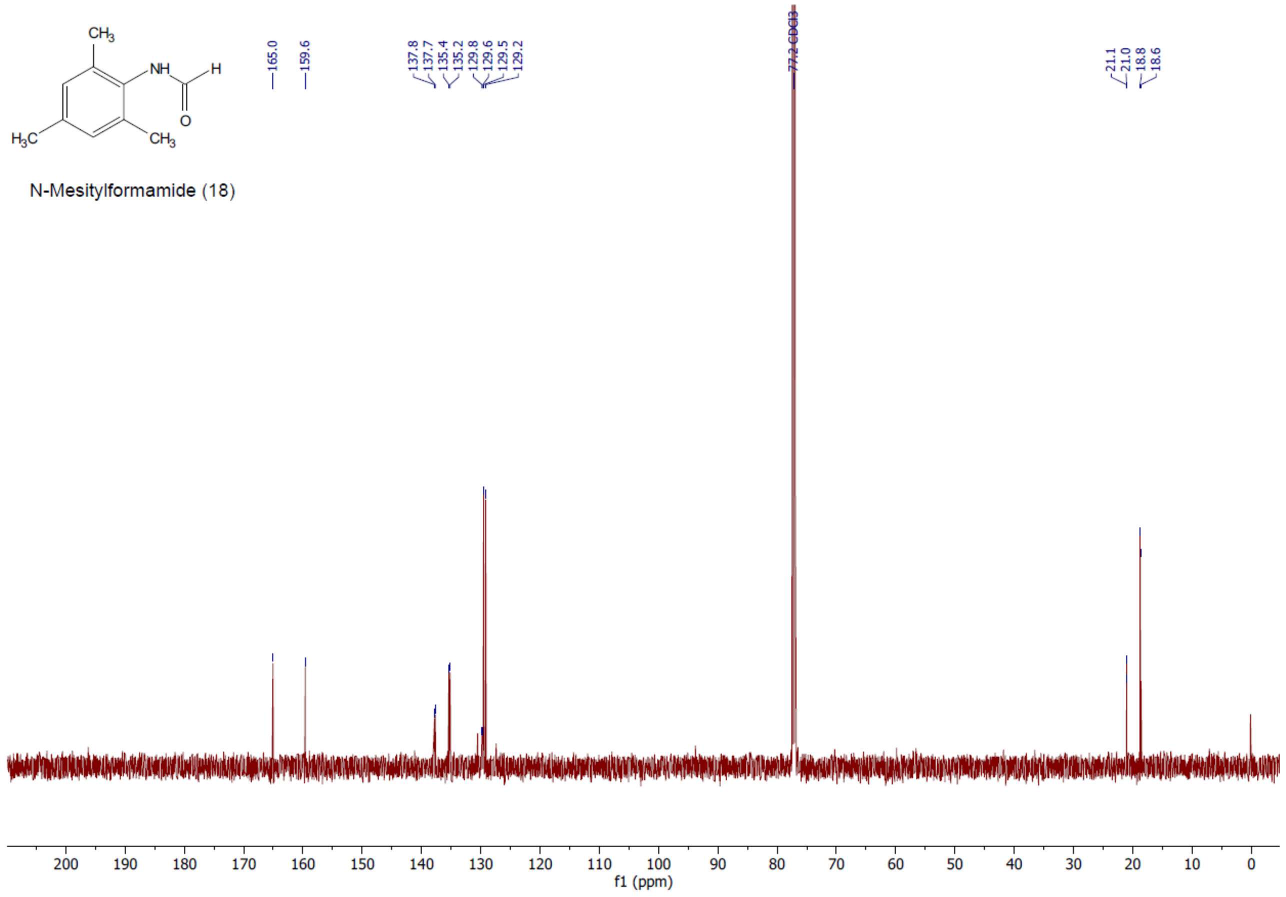

CH3
NH
H
O
H3C
CH3
N-Mesitylformamide (18)
165.0
159.6
137.8
137.7
135.4
135.2
129.8
129.6
129.5
129.2
77.2 CDCl3
21.1
21.0
18.8
18.6
f1 (ppm)

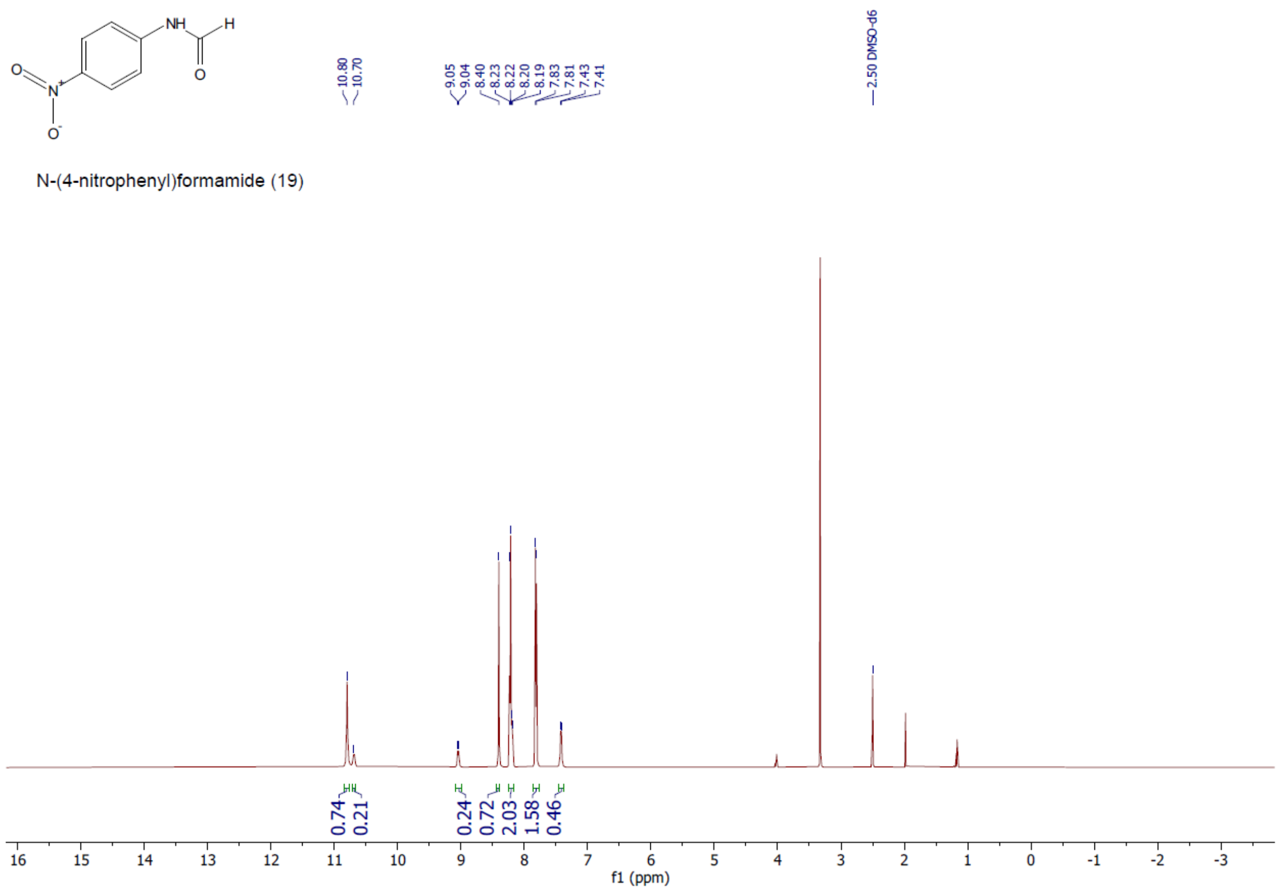
N-(4-nitrophenyl)formamide (19)
10.80
10.70
9.05
9.04
8.40
8.23
8.22
8.20
8.19
7.83
7.81
7.43
7.41
2.50 DMSO-d6
0.74
0.21
0.24
0.72
2.03
1.58
0.46
f1 (ppm)

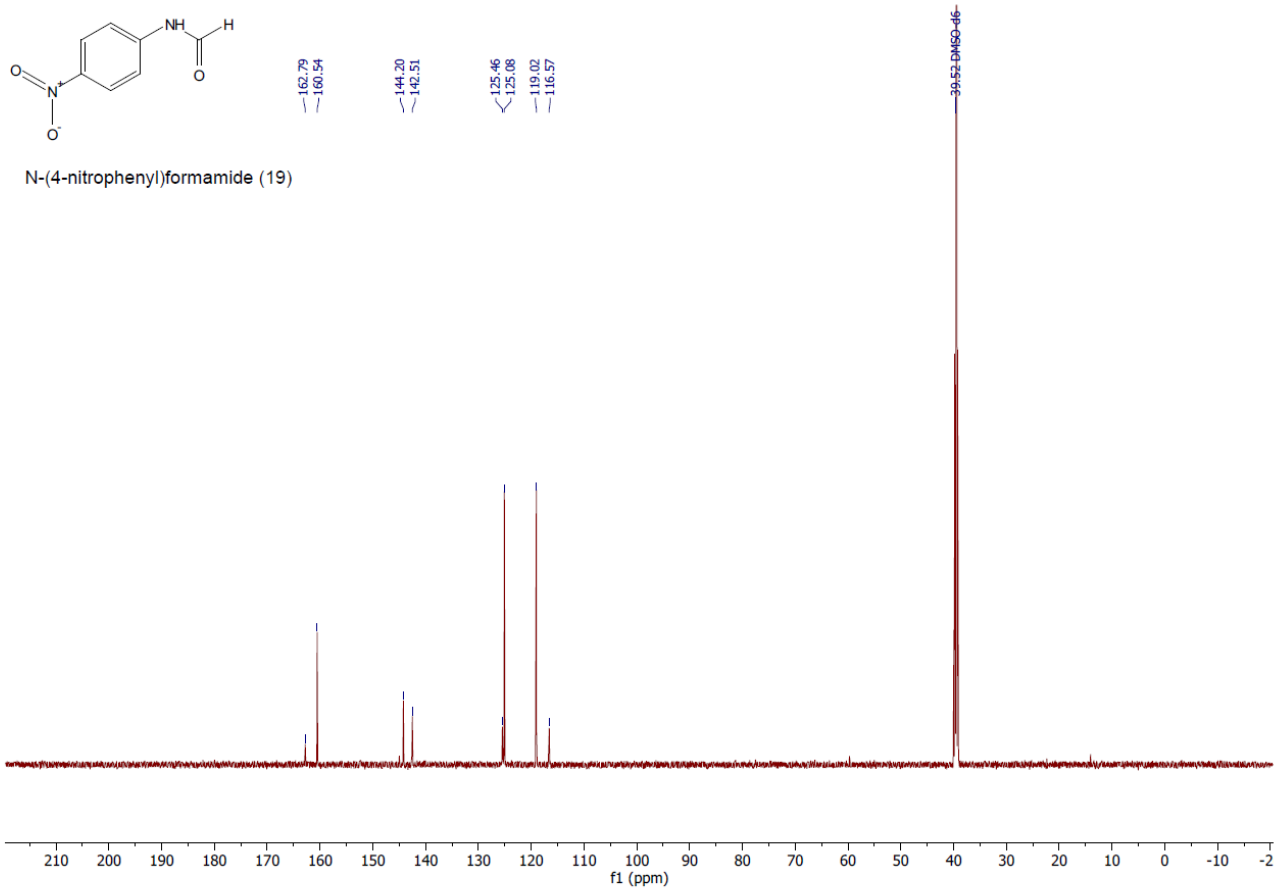
N-(4-nitrophenyl)formamide (19)
162.79
160.54
144.20
142.51
125.46
125.08
119.02
116.57
39.52 DMSO-d6
f1 (ppm)

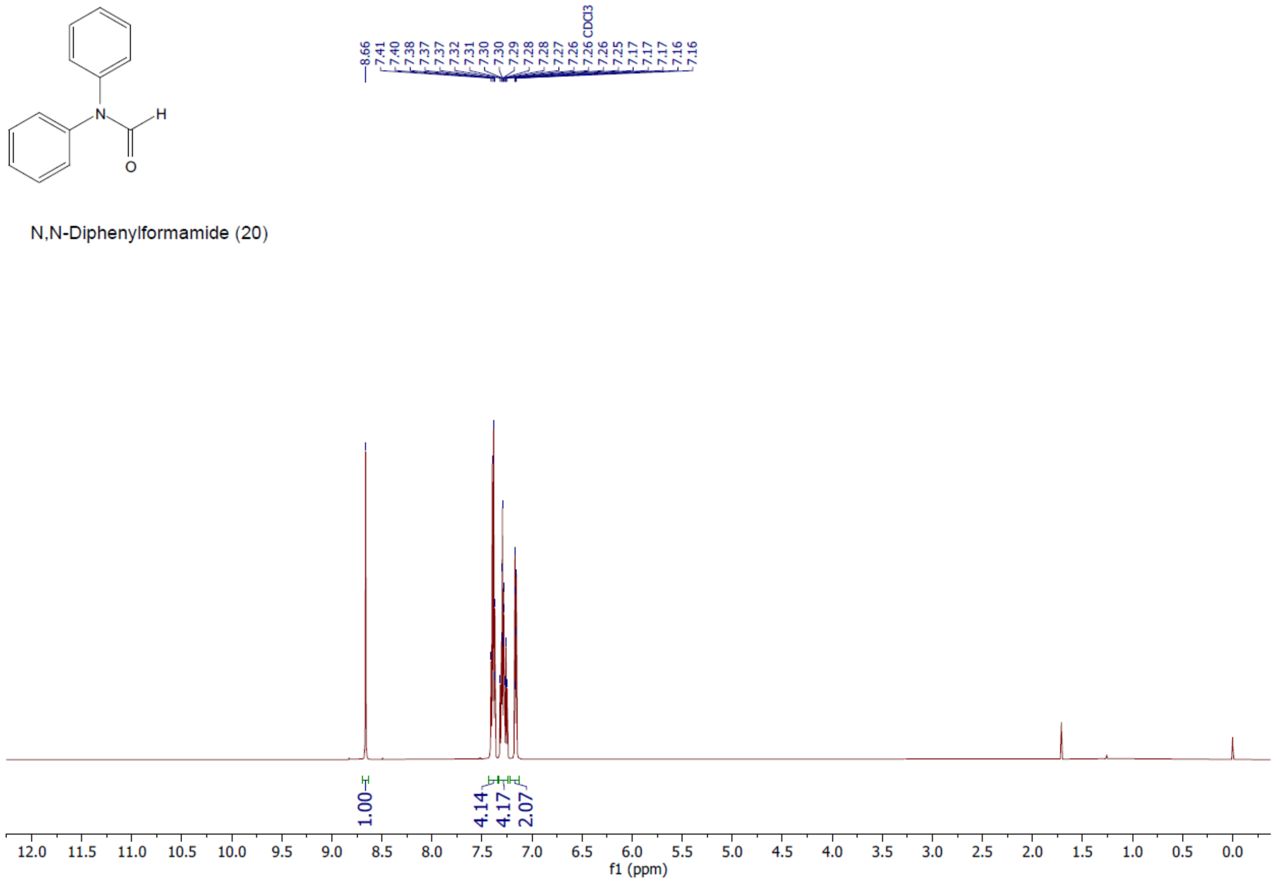

N,N-Diphenylformamide (20)

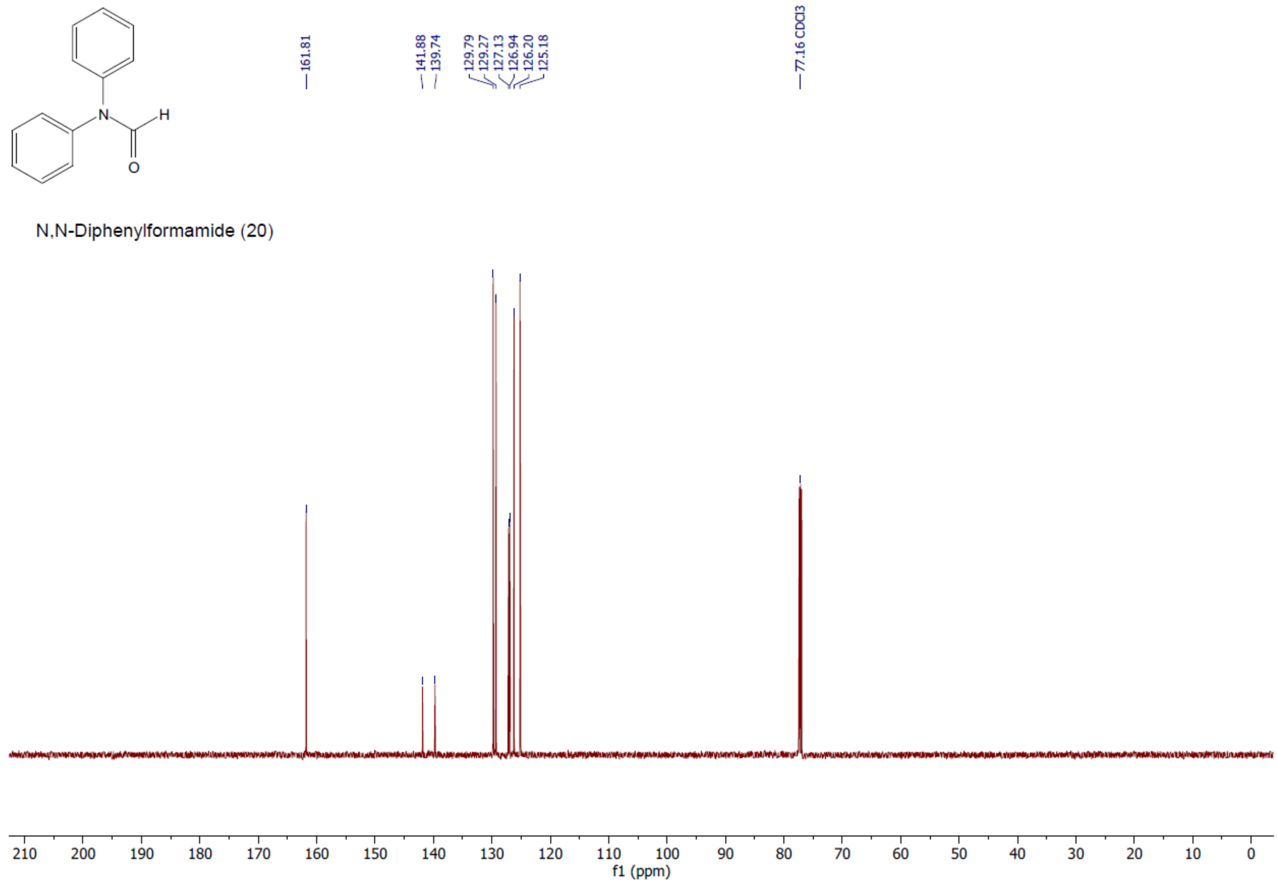

N,N-Diphenylformamide (20)

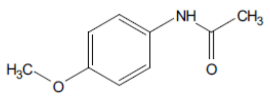

N-(4-methoxyphenyl)acetamide (21)

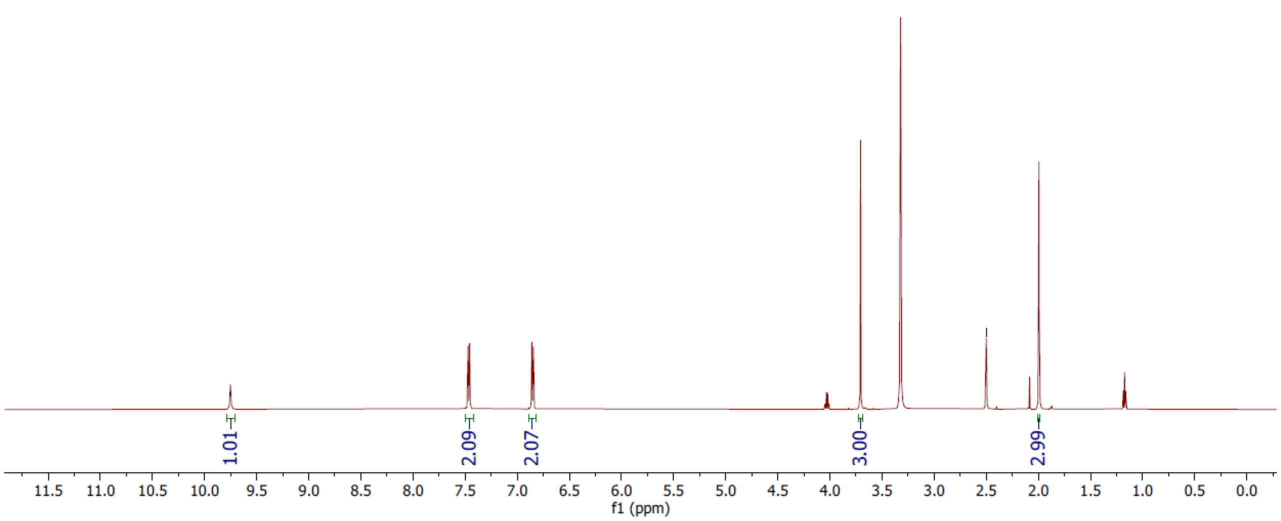

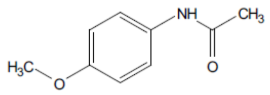

N-(4-methoxyphenyl)acetamide (21)

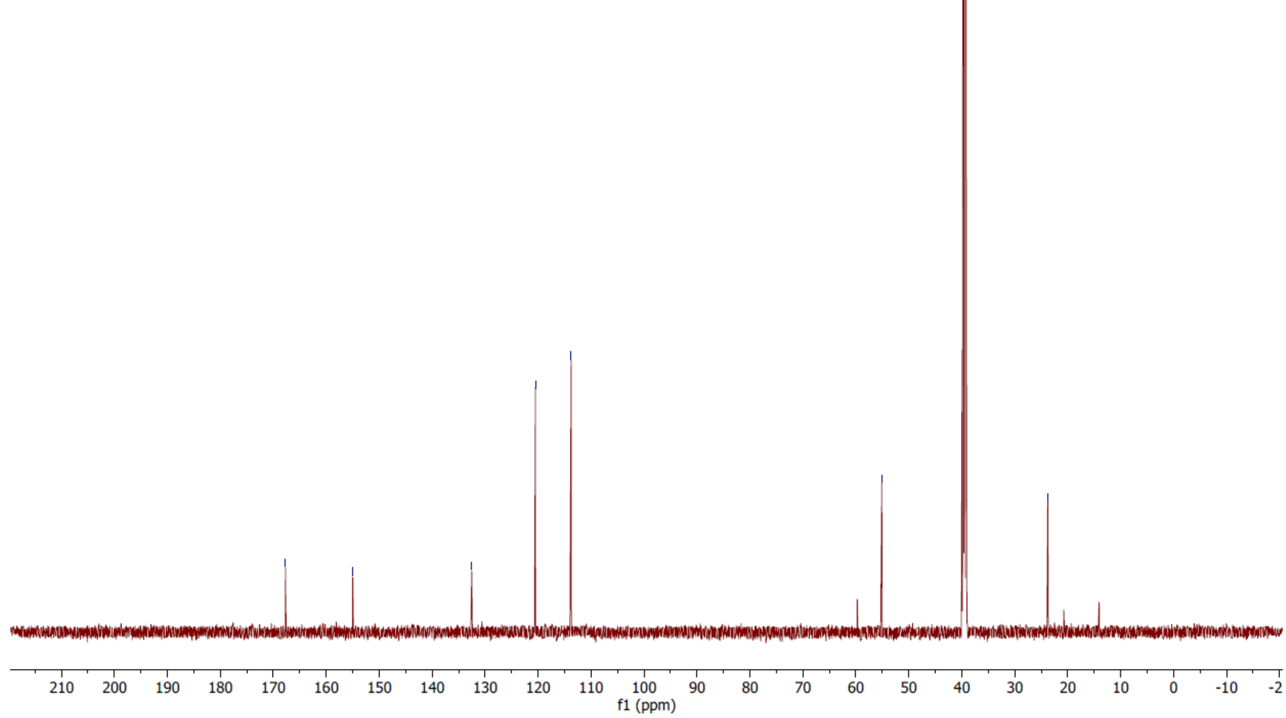

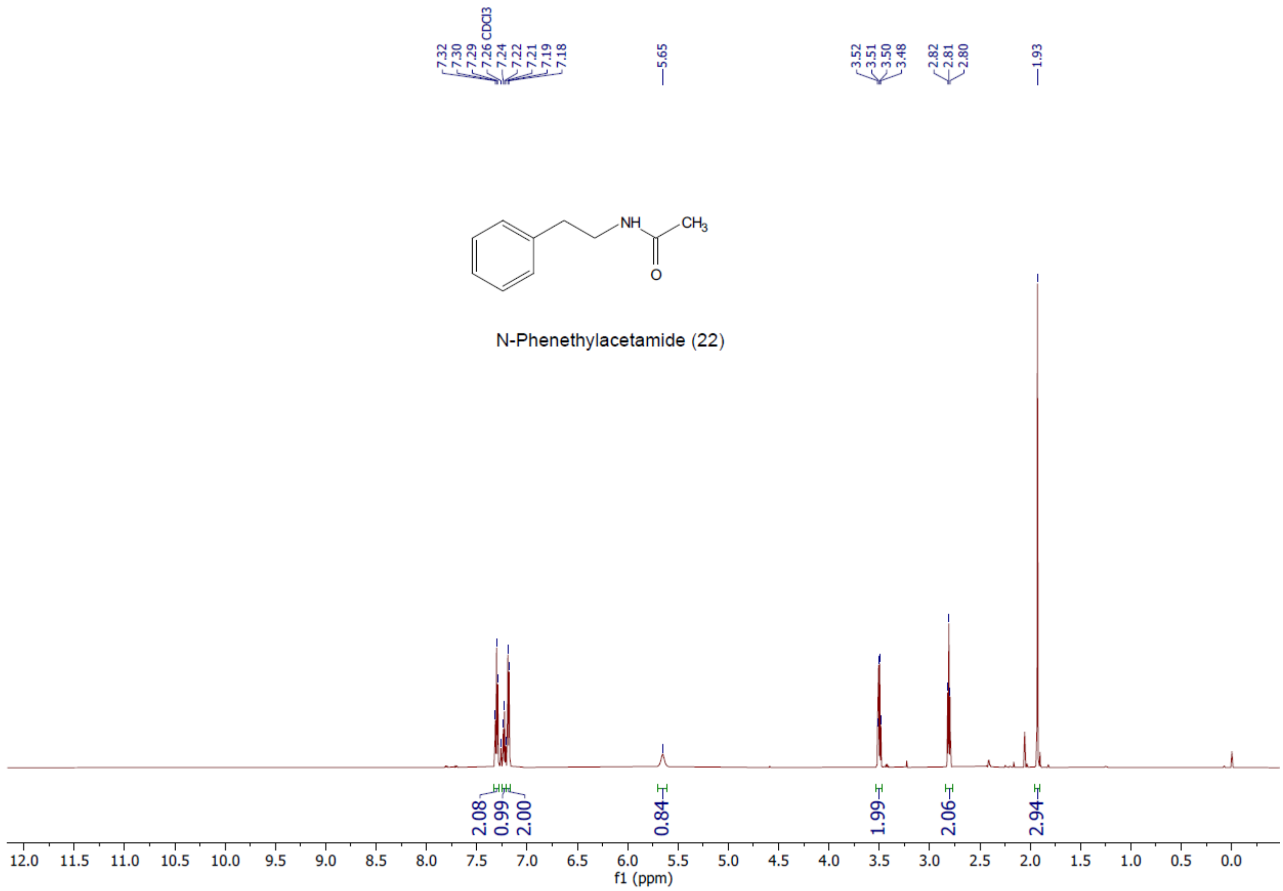

7.32
7.30
7.29
7.26 CDCl3
7.24
7.22
7.21
7.19
7.18
5.65
3.52
3.51
3.50
3.48
2.82
2.81
2.80
1.93
NH
CH3
O
N-Phenethylacetamide (22)
2.08
0.99
2.00
0.84
1.99
2.06
2.94
12.0 11.5 11.0 10.5 10.0 9.5 9.0 8.5 8.0 7.5 7.0 6.5 6.0 5.5 5.0 4.5 4.0 3.5 3.0 2.5 2.0 1.5 1.0 0.5 0.0
f1 (ppm)

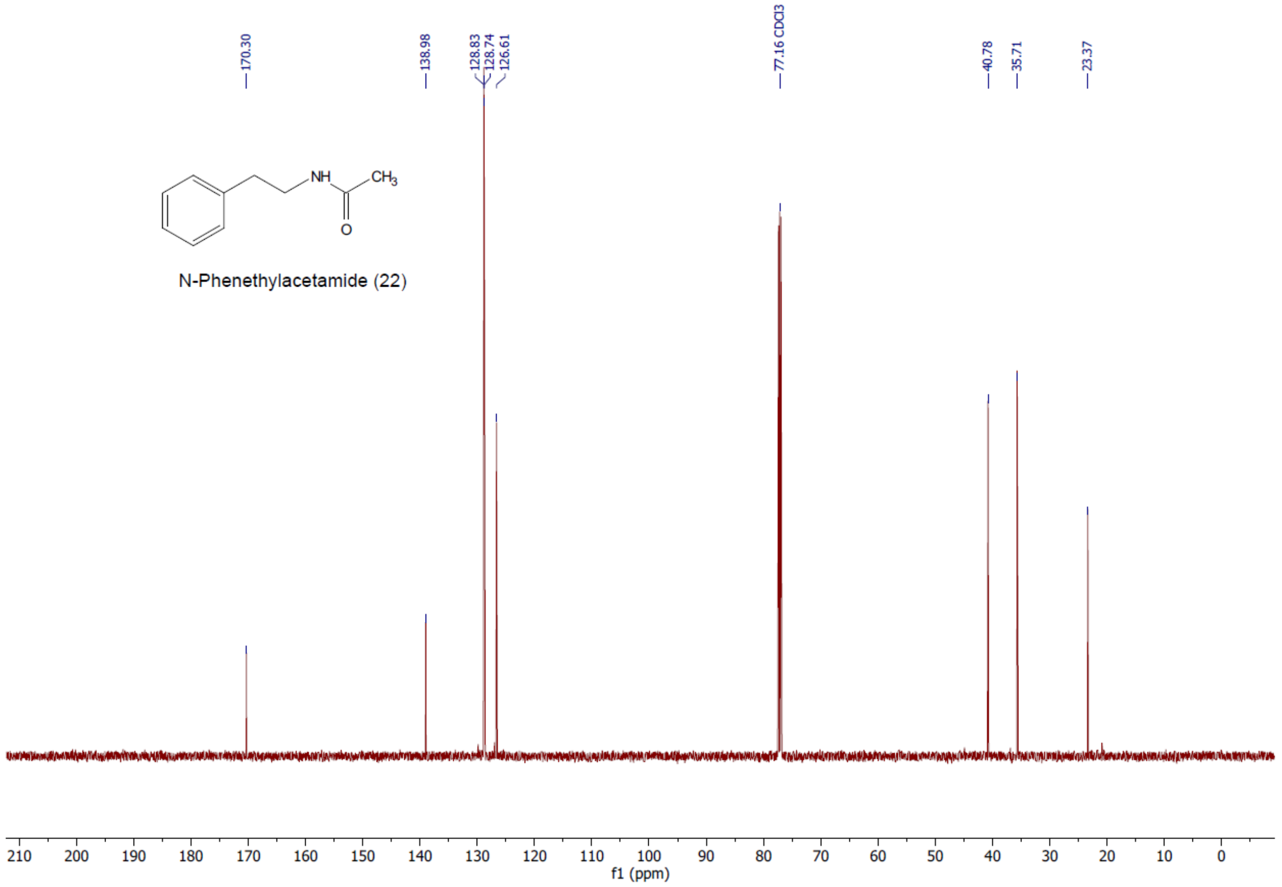

170.30
138.98
128.83
128.74
126.61
77.16 CDCl3
40.78
35.71
23.37
NH
CH3
O
N-Phenethylacetamide (22)
210 200 190 180 170 160 150 140 130 120 110 100 90 80 70 60 50 40 30 20 10 0
f1 (ppm)

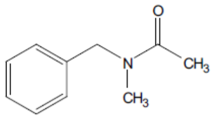

N-benzyl-N-methylacetamide (23)

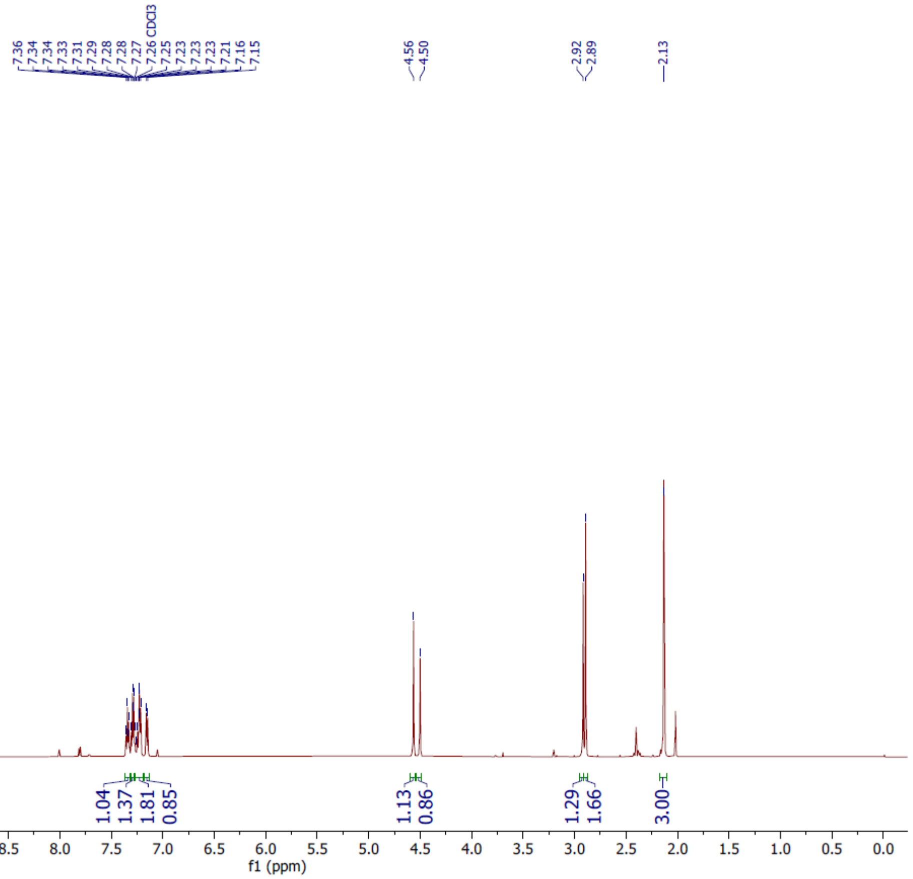

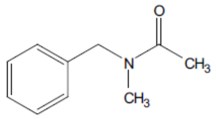

N-benzyl-N-methylacetamide (23)

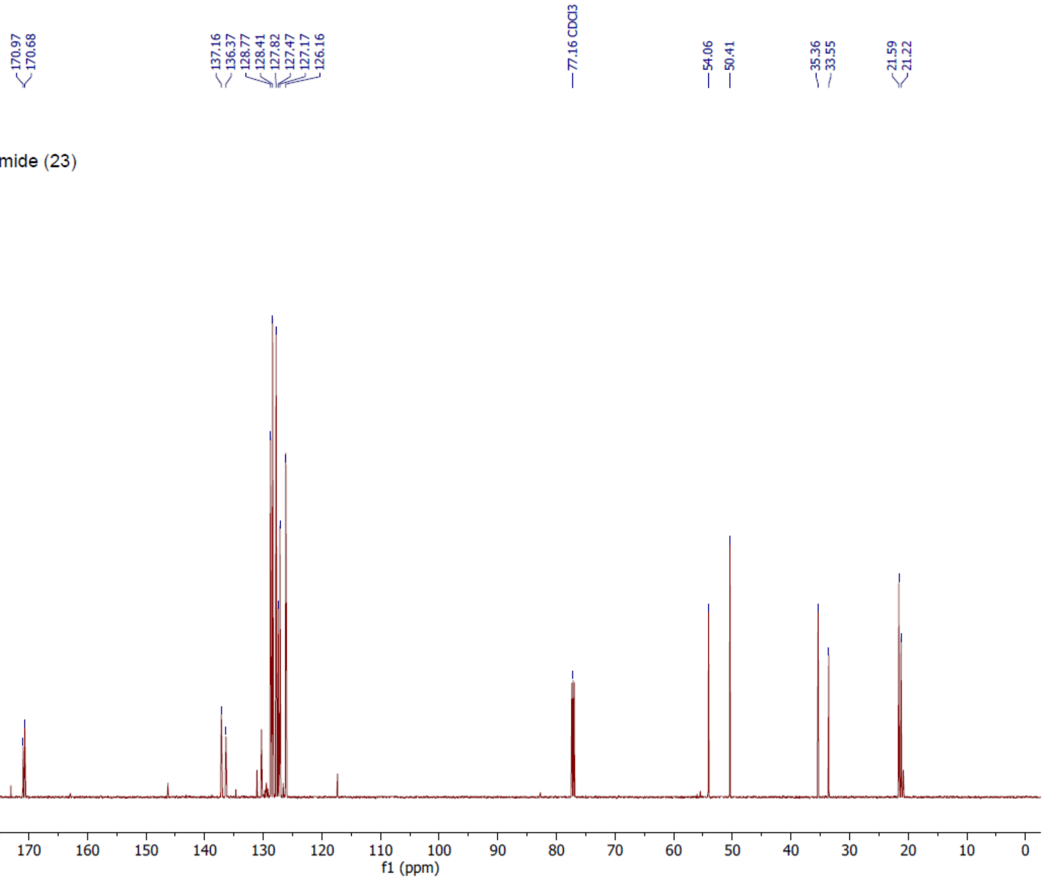

Supplement: File 1 — Experimental section and characterization of synthesized compounds. [file Beilstein_J_Org_Chem-18-1210-s001.pdf]
